# Behavioral and Neuroanatomical Consequences of Cell-Type Specific Loss of Dopamine D2 Receptors in the Mouse Cerebral Cortex

**DOI:** 10.3389/fnbeh.2021.815713

**Published:** 2022-01-13

**Authors:** Gloria S. Lee, Devon L. Graham, Brenda L. Noble, Taylor S. Trammell, Deirdre M. McCarthy, Lisa R. Anderson, Marcelo Rubinstein, Pradeep G. Bhide, Gregg D. Stanwood

**Affiliations:** ^1^Department of Biomedical Sciences, Florida State University College of Medicine, Tallahassee, FL, United States; ^2^Center for Brain Repair, Florida State University College of Medicine, Tallahassee, FL, United States; ^3^Instituto de Investigaciones en Ingeniería Genética y Biología Molecular, Consejo Nacional de Investigaciones Científicas y Técnicas and Universidad de Buenos Aires, Buenos Aires, Argentina

**Keywords:** D2 receptor, *DRD2*, conditional knockout, MK-801, motor learning, parvalbumin, interneuron, cerebral cortex

## Abstract

Developmental dysregulation of dopamine D2 receptors (D2Rs) alters neuronal migration, differentiation, and behavior and contributes to the psychopathology of neurological and psychiatric disorders. The current study is aimed at identifying how cell-specific loss of D2Rs in the cerebral cortex may impact neurobehavioral and cellular development, in order to better understand the roles of this receptor in cortical circuit formation and brain disorders. We deleted D2R from developing cortical GABAergic interneurons (*Nkx2.1*-Cre) or from developing telencephalic glutamatergic neurons (*Emx1*-Cre). Conditional knockouts (cKO) from both lines, *Drd2*^fl/fl^, *Nkx2.1-Cre*^+^ (referred to as GABA-D2R-cKO mice) or *Drd2*^fl/fl^, *Emx1-Cre*^+^ (referred to as Glu-D2R-cKO mice), exhibited no differences in simple tests of anxiety-related or depression-related behaviors, or spatial or nonspatial working memory. Both GABA-D2R-cKO and Glu-D2R-cKO mice also had normal basal locomotor activity, but GABA-D2R-cKO mice expressed blunted locomotor responses to the psychotomimetic drug MK-801. GABA-D2R-cKO mice exhibited improved motor coordination on a rotarod whereas Glu-D2R-cKO mice were normal. GABA-D2R-cKO mice also exhibited spatial learning deficits without changes in reversal learning on a Barnes maze. At the cellular level, we observed an increase in PV+ cells in the frontal cortex of GABA-D2R-cKO mice and no noticeable changes in Glu-D2R-cKO mice. These data point toward unique and distinct roles for D2Rs within excitatory and inhibitory neurons in the regulation of behavior and interneuron development, and suggest that location-biased D2R pharmacology may be clinically advantageous to achieve higher efficacy and help avoid unwanted effects.

## Introduction

The formation and subsequent function of the mammalian forebrain rely on the complex interplay of genetic and environmental factors through protracted periods of gestational and postnatal development. The assembly of brain circuitry occurs through an intricate continuum of processes, which change over time and which are highly dependent upon previous events to ultimately produce a normal functional state. Proliferation, cell migration, morphological and biochemical differentiation, and circuit formation all, therefore, rely on complex relationships between the extracellular environment and intracellular signaling that control particular developmental processes. Abnormalities in these processes contribute to multiple neurological and psychiatric disorders, often with symptom onset much later than the induction of pathology (Weinberger, 1987; Raedler et al., 1998; Rice and Barone, 2000; Lewis and Levitt, 2002; Andersen and Navalta, 2004). Thus, a constellation of genetic and environmental vulnerabilities can contribute to alterations in trajectories of brain development, with clinical symptoms apparent only later in life. In this regard, a wide range of neuropsychiatric disorders, including autism spectrum disorder, substance-use disorders, attention deficit hyperactivity disorder, and schizophrenia result from altered neuronal structure, function, and/or connectivity due to changes in neurodevelopmental trajectory (Finlay, 2001; Lewis and Levitt, 2002; Frederick and Stanwood, 2009; Thompson et al., 2009; Rapoport et al., 2012; Ben-Ari, 2013).

The early prenatal appearance, prior to synaptogenesis, of dopamine (DA) and other neuromodulators, led to early hypotheses concerning their role in brain development, and it is now recognized that neurotransmitters exhibit activities beyond classical synaptic transmission and modulation (Lauder, 1993; Whitaker-Azmitia et al., 1996; Levitt et al., 1997). Disruption of these signaling pathways during development can lead to permanent alterations in neuronal signaling, brain architecture, and behavioral outcome, depending on the time at which disruption occurs. Both genetic and environmental modulation of developing neurotransmitter systems produce complex and long-lasting effects on brain function (Malanga and Kosofsky, 1999; Gross et al., 2002; Maccari et al., 2003; Ansorge et al., 2004; Stanwood and Levitt, 2004; Bhide, 2009; Frederick and Stanwood, 2009; Thompson and Stanwood, 2009). Particularly within the prefrontal cortex, pre- and post-synaptic components of DA signaling exhibit development-specific changes across the lifespan (Rothmond et al., 2012).

Within the DA system, tyrosine hydroxylase (the rate-limiting enzyme in DA synthesis), DA receptors, and signaling proteins are expressed by embryonic day 14 in the rodent forebrain (Guennoun and Bloch, 1991, 1992; Jung and Bennett, 1996; Nakamura et al., 2000; Ohtani et al., 2003; Araki et al., 2007). Functionally, cell cycle length within neuroepithelial precursors of the forebrain is modulated by DA receptor activation (Ohtani et al., 2003; Popolo et al., 2004; Zhang et al., 2005). The migration of GABAergic interneurons to the cerebral cortex is also affected by DA receptor activation or genetic deletion (Crandall et al., 2007; Ohira, 2019). More globally, perturbations that alter DA receptor activation during development, including disruption of DA synthesis (Kim et al., 2000), neonatal lesion (Joyce et al., 1996; Neal-Beliveau and Joyce, 1999; Krasnova et al., 2007), or repeated DA agonist exposure early in postnatal life (Kostrzewa et al., 2008, 2016; Maple et al., 2015), produce brain region- and cell type-specific changes in receptor function and behavior. Over-expression of the D2R selectively in the striatum produces long-lasting changes in frontal cortex neurochemistry, D1 receptor (D1R) signaling, and working memory, even after the transgene has been switched off, indicating that the dysfunctions result from excess D2R activation during development (Kellendonk et al., 2006). *in vitro*, DA can serve as both a negative or positive regulator of neurite outgrowth, depending on cell type and the specific receptor(s) activated (Reinoso et al., 1996; Schmidt et al., 1996, 1998; Song et al., 2002; Stanwood and Levitt, 2007). *In vivo*, increases in dendritic length of the apical dendrites of pyramidal neurons and altered interneuron differentiation patterns are observed developmentally within the anterior cingulate cortex (ACC) of the D1R knockout (KO) mouse (Stanwood et al., 2005) and following pharmacological down-regulation of D1R signaling after prenatal cocaine (Stanwood et al., 2001; Stanwood and Levitt, 2007).

The frontal cortex, including the ACC, receives prominent DA innervation and expresses specific DA receptors in cell-type-specific patterns (Bjorklund and Dunnett, 2007; Tritsch and Sabatini, 2012; Roeper, 2013). Many successful pharmacological treatments for neuropsychiatric disorders target DA receptors, transporters, and intracellular signaling components (Niciu et al., 2013; Li et al., 2016; Ashok et al., 2017; Faraone, 2018; Alexopoulos, 2019). The D2R is particularly implicated in these actions (Ashok et al., 2017; Pergola et al., 2017; Selvaggi et al., 2019). However, there are limitations in our understanding of D2R signaling and its effect on brain circuitry function and structure, which may be explained in part by opposing functions of D2Rs in the two major neuronal subtypes within the cerebral cortex: excitatory glutamatergic pyramidal neurons and inhibitory GABAergic interneurons. As previously demonstrated by our group, D2Rs play a role in determining PV+ interneuron number and distribution in the frontal cortex (Graham et al., 2015b). This interneuron subpopulation is crucial in the regulation of synchronized neuronal activity (Cardin, 2018), and dysregulation of PV+ interneurons has been linked previously to the pathophysiology of schizophrenia, bipolar illness, and substance abuse (Rotaru et al., 2012). D2Rs also functionally modulate pyramidal neurons (Gee et al., 2012; Robinson and Sohal, 2017). We, therefore, undertook studies to explore the differential effects of genetic deletion of *Drd2* in cerebral cortical projection neurons and interneurons, to complement other recent studies in which D2Rs have been deleted in different subpopulations of neurons, including striatal medium spiny neurons (Durieux et al., 2009; Kharkwal et al., 2016), cells expressing Wolfram syndrome 1 (Puighermanal et al., 2020), DA neurons themselves (Bello et al., 2011; Holroyd et al., 2015), and parvalbumin-expressing interneurons (Tomasella et al., 2018). We observed distinct long-lasting neurobehavioral and cellular effects of deletion of D2Rs in these cell subpopulations.

## Materials and Methods

### Animals

We targeted deletion of D2Rs in either cortical GABAergic interneurons or cortical glutamatergic neurons from the frontal cortex as a cell type-specific knockout by crossing the D2R floxed mice (*Drd2*^fl/fl^) with either *Nkx2.1*-Cre or *Emx1*-Cre mice respectively (referred to as GABA-D2R-cKO or Glu-D2R-cKO mice here onwards). *Drd2*^fl/fl^ mice on a C57BL/6J background were kindly donated by Dr. Marcelo Rubinstein, which are now available in Jackson Laboratories (*Drd2*^tm1.1Mrub^, Stock #:020631, Bar Harbor, ME; Bello et al., 2011). Homozygous transgenic *Nkx2.1*-Cre mice (Tg(*Nkx2–*1-Cre)2Sand or BAC-*Nkx2.1*-Cre; Xu et al., 2008) and homozygous *Emx1*-Cre knockin mice (*Emx1*^tm1(cre)Krj^ or *Emx1*-IRES-Creknockin; Gorski et al., 2002) on a C57BL/6J background were obtained from Jackson Laboratories (Stock #:008661 and 005628 respectively). *Drd2*^fl/fl^ mice were bred with either *Nkx2.1*-Cre (Cre^+/+^) or *Emx1*-Cre (Cre^+/+^) mice to obtain *Drd2* ^fl/wt^, Cre^+/−^ offspring. These double heterozygotic offspring were bred to homozygotic floxed mice (*Drd2*^fl/fl^, Cre^−/−^) to obtain littermate *Drd2*^fl/fl^, Cre^+/−^ (*Drd2* conditional knockout; D2R-cKO) and *Drd2*^fl/fl^, Cre^−/−^ (*Drd2* homozygous flox; double flox (dFlox) control). As an additional control, the Cre mice (*Drd2*^wt/wt^, Cre^+/+^) were bred to wild-type (WT) mice, and the offspring were used for testing (*Drd2*^wt/wt^, cre^+/−^; Cre control). *Drd2*^fl/fl^ (dFlox) controls and D2R-cKO mice were bred as littermates, whereas Cre controls were non-littermates from both dFlox controls or D2R-cKO mice. In order to map patterns of Cre-induced recombination, the Cre lines were also crossed to B6.Cg-Gt(ROSA)26Sortm14(CAG-tdTomato)Hze/J mice (Stock #:007914, Jackson Laboratories, Bar Harbor, ME, USA).

Offspring were kept with the dam and sire until weaning at postnatal day 21 (P21) when they were separated into same-sex cages and housed with littermates until experimentation at adulthood (P60+). Adult mice were housed 3–5/cage and were provided with rodent chow and tap water *ad libitum*. Mice were housed in a temperature- and humidity-controlled Association for Assessment and Accreditation of Laboratory Animal Care International-approved facility that is maintained on a 12:12 h light/dark cycle (lights on 0600−1800 h). Both males and females were utilized for all histological and behavioral assays. No obvious differences in histological or molecular indices were observed between male and female mice. Sex differences in some behavioral responses are reported below. All lines were fully backcrossed (>10 generations) to a C57BL/6J background. All protocols were approved by the Florida State University Animal Care and Use Committee (ACUC) and all studies were performed in accordance with the recommendations in the National Institute of Health’s Guide for the Care and Use of Laboratory Animals. Behavioral experiments were performed in 9- to 12-week-old mice during the light cycle (0600 and 1800 h). Unless otherwise noted, all experiments were completed and tissue samples were harvested at adulthood (P60+) to ensure proper age-matched controls. Genotypes were confirmed by the polymerase chain reaction analysis of the tail tissue obtained at weaning, which were reconfirmed at death.

### Neurobehavioral Paradigms

Mice from each of the genotypes (D2R-cKO, dFlox control, and Cre control) were used in behavioral studies. Male and female mice (P60–140; *n* = 5–8/genotype) were tested in a behavioral battery consisting of elevated zero maze (EZM), light/dark test, Y-maze, novel object recognition (NOR) test, forced swim test (FST), rotarod, novelty-induced open field test, and open field test with MK-801 or amphetamine challenge; multiple cohorts of animals were used to complete behavioral tests. Mice were age-matched for each behavioral test, were weighed at the start of each test, and only one test was performed per day with at least 24 h in between each test. Mice were extensively handled for at least three consecutive days prior to the initiation of the testing battery. Each apparatus used for testing was cleaned with 1.6% quatricide spray between animals.

### Drugs

(+)-MK-801 maleate (dizocilpine; Abcam, Cat: Ab120027, Cambridge, UK) and amphetamine (National Institute for Drug Abuse, Bethesda, MD, USA) were injected intraperitoneally at doses of 0.3 and 3 mg/kg, respectively, for behavioral pharmacology studies.

### Open Field Test for Spontaneous and Drug-Stimulated Locomotor Activity

Both novelty-induced locomotor activity and drug-induced locomotor responses were measured using commercial open field activity chambers (Med Associates, 29 × 29 × 20.5 cm). These open field activity chambers were contained within light- and air-controlled environmental chambers (Med Associates, St. Albans, VT; 64 × 45 × 42 cm; Killinger et al., 2012; Graham et al., 2015a). All movements were analyzed with the Med Associates Activity Monitoring program, as measured by beam breaks on the x-y-z axes (16 infrared beams, 50 ms intervals). Ambulatory distance (cm) and time, as well as time spent in the center or surround zones (i.e., thigmotaxis), were analyzed within 5-min blocks. Ambulatory distance (cm) as a measure of distance traveled was averaged between two sessions performed for each mouse and was grouped by genotype. To test for basal locomotor activity, locomotor responses were recorded for 60-min in the open field arena. The ambulatory distance traveled was then reported as an average for the testing session for the basal locomotor activity. For drug challenge, a 3-day protocol was employed as previously described (Frederick et al., 2012); on days 1 and 2 of testing, mice were placed into activity chambers for 30-min for baseline measurements. Post-habituation to the chamber, mice were removed from the chambers, injected with 0.9% saline, and returned back to the chambers for an additional 120-min for a total of 150-min testing session in the open field arena. On day 3, mice were injected intraperitoneally with MK-801 (0.3 mg/kg), amphetamine (3 mg/kg), or control (0.9% saline). Distance traveled within 5-min blocks was averaged by genotype for the full 150-min testing duration. Animals were not used in any other tests or for tissue collection following drug administration.

### Elevated Zero Maze

EZM was used to measure anxiety-like and risk-taking behaviors, as mice are more willing to spend time in closed arms than open ones. The EZM takes advantage of the natural tendency for mice to explore novel environments, while also being avoidant towards bright-lit, elevated, open space as compared to a dark, enclosed space. This approach-avoidance conflict results in behaviors and has been correlated with increased physiological stress or anxiety-like behavior (Holmes et al., 2003). Testing was performed as previously described (Frederick et al., 2012, 2015; Graham et al., 2015a). Briefly, mice were placed into an open area of an elevated circular platform (~6 cm width with a ~40 cm inner diameter) that is equally divided into four quadrants. Two quadrants on opposite sides of the platform are enclosed by walls (~20 cm high); the other two quadrants are open and bordered by a lip (~0.6 cm high). Mice were allowed to explore the apparatus for 5-min under red light conditions, and activity was monitored and recorded *via* an overhead camera connected to a computer using ANY-maze software (Stoelting, Wood Dale, IL, USA). Video recording was analyzed by the experimenter, blinded by genotype. The number of entries into the open arms and the amount of time spent in the open arms were used as measures of anxiety and other output measures included percent time spent in open or enclosed areas, entries into open areas, speed, and total distance traveled.

### Light/Dark Test

The light/dark test is based on the innate aversion of mice to brightly lit areas and their spontaneous exploratory behavior in response to mild stressors (novel environment and light; Crawley and Goodwin, 1980). Taking advantage of this innate aversion, the light/dark test is widely used to test for anxiety-like and risk-taking behaviors as mice are and test the animal’s willingness to explore an open, well-lit area instead of staying in the darkened, “safe” chamber. Mice were placed individually into the lighted compartment at the beginning of the session and the total time spent in the dark compartment and the number of light-dark transitions were measured during the 10-min testing session. All movements and animal locations were analyzed with the Med Associates Activity Monitoring program as previously mentioned. Ambulatory distance (cm) and the time spent in and latency to enter the darkened compartment were analyzed.

### Y-Maze

The Y-maze was used to test spatial memory and attention and was performed as previously described using a three-armed apparatus with distinct spatial cues at the end of each arm (Carpenter et al., 2012; Frederick et al., 2012; Graham et al., 2015a). As mice are more willing to explore a new arm of the maze as opposed to the one that it was just in prior, we recorded the number and pattern of arm choices as the mice are allowed to freely explore for up to 6-min. Briefly, the testing apparatus was constructed from clear Plexiglas consisting of three arms (24” × 6” × 12”) angled 120° apart in the shape of a “Y” with removable walls at the center end and joined by a central triangular platform. Visual cues were placed around the testing room. A mouse was placed into one of the three arms of the apparatus, and arm entries were recorded by an observer, and these data were used to calculate total arm entries and spontaneous alternation, which was defined as the consecutive entry into each of the three arms (Lalonde, 2002; Thompson et al., 2005; Gustin et al., 2011; Graham et al., 2015a). The number of possible alternations (# of total entries—2) was used to calculate percent spontaneous alternations [(# of spontaneous alternations/# of possible alternations) × 100]. Chance performance was defined as 22.2% in this paradigm as previously described (Thompson et al., 2005).

### Novel Object Recognition Test

The NOR test was used to test non-spatial working memory to assess memory between two objects as described previously (Thompson et al., 2005; Gustin et al., 2011; Killinger et al., 2012). The day before testing, mice were habituated to the empty novel object arena (39.5 × 28.5 × 19.5 cm) for 20-min. The following day, mice were placed into an arena with two identical objects placed on two sides of the open arena for 5-min. After a 10-min inter-trial interval in the home cage, mice were returned to the arena and allowed to explore two objects, one identical to the original objects and the other different, for an additional 5-min. Except for day 1 habituation, all sessions were monitored and recorded *via* an overhead camera connected to a computer using ANY-maze software (Stoelting). Time spent exploring novel vs. familiar objects was recorded *post hoc* video analysis, experimenter blinded by genotype. A counter-balanced design was used to control for the side of presentation and side in which the novel object was placed as well as which object was used as the familiar and novel objects to prevent side or object bias, respectively. For data analysis, time spent with the novel object and the familiar object was calculated as percent preference (time interacting with novel object/time interacting with novel + familiar objects).

### Forced Swim Test

The forced swim test (FST) was used to measure depression-related behaviors as affected mice struggle less and/or become immobile sooner than control mice when placed in an inescapable environment. Mice were placed in plastic cylinders (50 cm in diameter, 21 cm in height) filled approximately 3/4th full with room temperature water so that they are not able to escape from the beaker or touch the bottom. Mice were individually placed into the cylinder for a 6-min test and were monitored and recorded for the duration of the testing session *via* a camera connected to a computer using ANY-maze software (Stoelting). The experimenter monitored the mouse during the task and if there was any indication that the mouse was struggling to keep its mouth above water or was in danger of drowning, it was removed from the beaker immediately and was excluded from the study. After testing, the mice were placed into a cage (~35–37°C) sitting on a heating pad to dry before returning to their home cage. The water was changed in between tests and the temperature of the water was recorded. Videos were later analyzed for the amount of time spent immobile for each mouse, which was calculated as percent immobility [(time spent immobile/total time spent inside beaker) × 100%]; the observer was blinded to genotype during analysis. Since the first 2-min of testing is commonly defined as habituation or pre-testing session, we assessed the percent immobility during both the full 6-min testing session (combining both pretesting and testing sessions) as well as during the last 4-min testing session (testing session; Yankelevitch-Yahav et al., 2015) as previously described (Frederick et al., 2012, 2015; Graham et al., 2015a).

#### Rotarod

The rotarod test is widely used to assess motor coordination and performance in mice (Zausinger et al., 2000; Luesse et al., 2001; Jeong et al., 2003; Karl et al., 2003). Thus, we used the rotarod to measure motor coordination and motor learning as measured using a standard rotarod apparatus, consisting of a cylinder (3 cm in diameter), which rotates at speeds ranging from 5 to 40 rpm as previously described using three 5-min trials per day with 15-min inter-trial interval over the course of three consecutive days (Frederick et al., 2012; Graham et al., 2015a). Mice were placed on an accelerating rotarod apparatus (Ugo Basile model 47650; Collegeville, PA, USA). The latency to fall was recorded, and values for the three daily trials were averaged each day.

#### Barnes Maze

Since frontal cortical circuits are known to be involved in cognitive function required for proper spatial working memory and behavioral flexibility, we wanted to further assess changes in spatial working memory and reversal learning through a Barnes maze (Sutherland et al., 1988; Dias et al., 1996, 1997; Kesner et al., 1996; McCarthy et al., 2018). Unlike other similar tests such as Morris Water Maze or radial-arm maze, Barnes maze is known to be less physically taxing and less stressful since a weak aversive stimulation (light, fan) is used to increase the motivation for the mice to escape the maze (Markowska et al., 1998; O’Leary and Brown, 2009). The maze apparatus was 122-cm in diameter and 140-cm high and was designed with 40 equally spaced holes on the periphery of the surface. Visual cues arranged in the room around the maze served as spatial cues. A dark box filled with bedding was positioned under one of the holes on the platform to allow the mouse to escape from aversive stimuli, which are a high intensity fan blowing air at the level of the platform and two 120-watt flood lamps hung from the ceiling immediately above and aimed at the platform. Each mouse was assigned an escape hole for the duration of the experiment. For each trial, the mouse was placed in the center of the platform facing away from the target escape hole. Each mouse was trained to find the dark box with two 4-min trials per day for nine consecutive days (acquisition training). The inter-trial interval was typically 20-min. Each trial ended when the mouse entered the escape hole; the experimenter then returned the mouse to its home cage ~10-s later. If the mouse did not find and enter the escape hole in 4-min, the experimenter picked up the mouse and placed it inside the escape hole and assigned the maximum time value for the latency to escape (4-min). During this period, the search strategy utilized to escape was also recorded as either random (two or more crossings through the maze center; a nonsystematic approach), serial (crosses the maze less than two times and explores adjacent holes to find the escape), or spatial [no crosses over the maze center and explores within the general vicinity (±4 holes) of the escape hole]. The day after the completion of acquisition training, the mice were given a probe trial, which consisted of one 5-min session on the maze, with no escape box. Since this probe trial was essentially an extinction session, mice were then re-trained back to the location assigned during acquisition training on the following day with an escape hole box, with two 4-min trials as described above. Next, the location of the escape hole was switched to the location diagonally opposite (180°) the initial position. Mice were tested for their ability to find the reversed escape hole with two 4-min trials per day over the subsequent 3–5 days (reversal phase) to test for behavioral flexibility. Each trial was monitored and recorded with an overhead video camera connected to a computer *via* ANY-maze software (Stoelting). The video was coded by an experimenter blinded by genotype for primary latency (s) and primary error (#), with measurements averaged between two trials for each animal.

#### Tissue Preparation

For immunohistochemistry (IHC), adult D2R-cKO mice (P70-P120, *n* = 6–9/genotype) or ROSA reporter-mice (P60–90, *n* = 4 per line) were anesthetized with sodium pentobarbital and transcardially perfused with 4% paraformaldehyde (PFA). Brains were removed and fixed overnight in PFA at 4°C. Following the cryoprotection method in a sucrose gradient (10%, 20%, and 30% in phosphate-buffered saline), brains were cut coronally into 40 μm sections on a freezing microtome and stored at −20°C in the freezing solution until further experimentation. For *in situ* hybridization (ISH), a separate cohort of adult mice (P70-P120, *n* = 4–6/genotype) was anesthetized with isoflurane and decapitated, where whole brains were removed and immediately flash frozen in cold, 2-methylbutane. Samples were cut coronally into 20 μm sections on a cryostat (maintained temperature at −20°C) directly onto Superfrost Plus Microscope slides (Cat: 12-550-15, Fisher, Pittsburgh, PA), and brain sections were cut approximately +0.98 − +1.98 mm from Bregma, focusing on the frontal cortex in a series of ten, with each slide containing four non-adjacent brain sections. Slides were then air-dried for 30-min at room temperature before being used immediately for ISH experiment or placed in storage at −80°C until experimentation.

#### *In situ* Hybridization and Fluorescent Immunohistochemistry

For the ISH experiment, we used the RNAScope^®^ Multiplex Fluorescent v2 assay with RNAScope^®^pProbes and the RNAScope^®^ Multiplex Fluorescent Reagent Kit v2 commercially available from Advanced Cell Diagnostics (ACD; Cat: 323100, ACD, Fresno, CA, USA). The RNAScope^®^ probes contain 20 ZZ probe pairs (50 bp/pair) typically covering ~1,000 bp. Probes against vesicular glutamate transporter 1 (vGLUT1; Slc17a7, NM_182993.2, Probe-Mm-Slc17a7, Cat: 416631, ACD), Gad2 (Gad2, NM_008078.2, Probe-Mm-Gad2-C3, Cat: 439371-C3, ACD), Drd2-E2 (Drd2-E2, NM_010077.2, Probe-Mm-Drd2-E2-C2 for the exon2-specific Drd2 gene, Cat: 486571-C2, ACD) were used. The assays were performed as previously described using fresh frozen tissue (Graham et al., 2020, 2021). Sections were counterstained using DAPI (1 μglmL, Cat: D9542, Sigma-Aldrich, St. Louis, MO). Images were visualized *via* a Zeiss AxioImager or Keyence BZ-X fluorescent microscope at 20–60× magnification. Additional select images were captured *via* an FV3000 confocal laser scanning microscope (Olympus, Tokyo, Japan) to verify the fidelity and specificity of channels and labels for colocalization studies.

ROSA reporter mice were sectioned and stained for fluorescent IHC as previously described (Jacobs et al., 2009). Mouse anti-PV (1:500 dilution, Cat: P3088, Sigma-Aldrich), rat anti-somatostatin (SST; 1:400 dilution, Cat: MAB354, Sigma-Aldrich, St. Louis, MO), goat anti-choline acetyltransferase (CHAT; 1:400 dilution, Cat: AB144P, Sigma-Aldrich, St. Louis, MO), or mouse anti-DARPP-32 (1:1,000 dilution, Cat: 611520, BD Biosciences, San Diego, CA) was incubated in free-floating brain sections for 72-h at 4°C, followed by a 1-hr incubation with Cy2-Affinipure donkey anti-mouse, anti-rat, or anti-goat secondary antibodies (1:1,000 dilution, Cat: 715-225-150, 712-225-150, and 705-225-147, respectively; Jackson ImmunoResearch, WestGrove, PA, USA). Sections were counterstained using DAPI (1 μg/ml, Cat: D9542, Sigma-Aldrich) and images were visualized *via* a Zeiss AxioImager or Keyence BZ-X fluorescent microscope.

#### Chromogenic Immunohistochemistry

Staining was performed as previously described using the chromogen 3,3’ diaminobenzidine (Stanwood et al., 2005, 2009). Monoclonal antibodies against GAD67 (1:2,000 dilution, Cat: MA5406, Millipore, Billerica, MA), PV (1:500 dilution, Cat: P3088, Sigma-Aldrich), and SST (1:400 dilution, Cat: MAB354, Sigma-Aldrich) were used with Biotin-SP donkey anti-mouse and anti-rat secondary antibodies (1:1,000 dilution, Cat: 715-065-150 and 712-065-150, respectively; Jackson ImmunoResearch, WestGrove, PA, USA). Sections were visualized using a Zeiss AxioImager microscope with a Zeiss AxioCam HRc camera and corresponding AxioVision 4.1 software. For GAD67 and PV cell counts, brain sections were selected from the ACC (approximately +0.98 to +1.98 mm from Bregma), with at least three sections from bilateral hemispheres photographed or observed, blinded to genotype. Sections were analyzed per region of interest × hemisphere by an observer blinded to genotype. For GAD67 and PV cell counts, sections were imaged at 20× as previously described (Graham et al., 2015b). For each hemisphere in each D2R-cKO line, two non-overlapping fields of ACC (dorsal and ventral portions) were counted. Immunoreactive profiles were also counted in the somatosensory cortex (SSC) from the GABA-D2R-cKO mice. For each region examined, cell counts were summed per section and corrected for profile size using the Abercrombie correction method (Abercrombie, 1946) and then calculated as densities (corrected counts/mm^2^), and images were counted and analyzed using ImageJ software. Sections from D2R-cKO and control mice were always immunostained in parallel within the same experiment.

#### Data Analysis and Statistics

Statistical analyses were performed using the GraphPad Prism 8 (GraphPad Software, San Diego, CA), with significance set at *p* ≤ 0.05 and unless stated otherwise, data are presented as mean ± standard error of the mean (SEM). The statistical significance of differences between means was assessed using the one- or two-way analysis of variance (ANOVA) at the 95% confidence interval, followed by Tukey’s multiple comparison *post hoc* testing. Outlier analysis was performed using the ROUT method with the false discovery rate set at 1%. Individual data points were considered outliers if their value was greater than 2 standard deviations above or below the mean for each trial and were thus eliminated for that particular trial or test. Histological data were analyzed *via* one-way ANOVA with a *post hoc* Tukey’s multiple comparison test with Genotype as the main factor. However, as we found no significant differences between the dFlox and Cre controls, we combined these values and utilized a Student’s *t*-test. For EZM, light/dark, FST, Y-maze, NOR test, rotarod, and open field tests, data were analyzed *via* two-way ANOVA (Genotype × Sex) with a *post hoc* Tukey’s multiple comparison test in order to compare mean differences. Where there was no statistically significant main effect of Sex, data were collapsed and subsequently re-analyzed by Genotype only. In cases where more than two main effects were analyzed (e.g., Sex, Genotype, Time), the PROC MIXED procedure in SAS (version 9.4; Cary, NC) was used. Significant interactions were further analyzed using slice-effect ANOVAs. Statistical significance was set at *p* < 0.05. The type of statistical analysis used was determined independently for each experiment and statistical analyses for each dataset are described in results and figure legends.

## Results

### Validation of Cerebral Cortical GABAergic- and Glutamatergic-Specific Deletion of D2R

**Figure 1:**
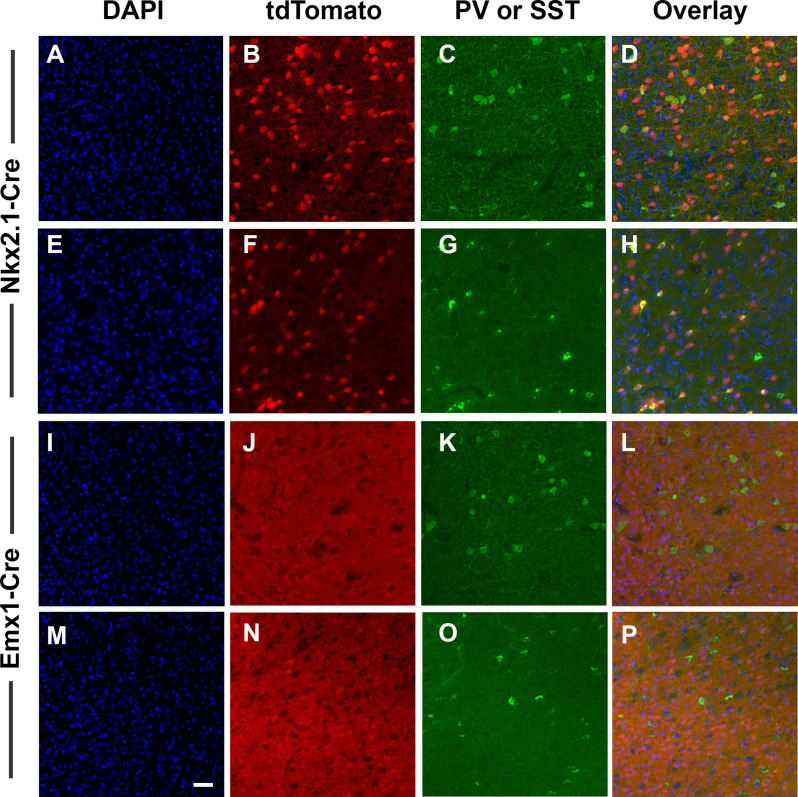
Validation of cell-type specificity of recombination. *Nkx2.1*-Cre and *Emx1*-Cre mice were bred to the B6.Cg-Gt(ROSA)26Sortm14(CAG-tdTomato)Hze line as an initial validation test. Offspring express bright tdTomato fluorescence throughout each cell where Cre-mediated recombination has occurred (red). Images were collected with a 20× objective and scale bar = 50 μm. The result of *Nkx2.1*-induced recombination in the anterior cingulate cortex (ACC) is shown in panels **(A–H)**, and following *Emx1*-Cre-induced recombination in panels **(I–P)**. Sections were also processed for DAPI (blue) and for immunostaining against PV (green **C,K**), or SST (green, **G,O**). *Nkx2.1*-Cre-induced recombination was present in scattered cells throughout the ACC **(B,F)**, and included interneurons immunoreactive for PV **(C)** and SST **(G)**. In contrast, *Emx1*-Cre-induced recombination was present in a dense array of cells and processes throughout the ACC **(J,N)**, but interneurons immunoreactive for PV **(K)** and SST **(O)** were largely absent of tdTomato, consistent with the identity of *Emx1*-Cre-derived recombined cells being glutamatergic pyramidal cells rather than GABAergic interneurons. Images displayed are representative of *N* = 3–5/genotype.

We targeted deletion of D2Rs from *Nkx2.1+* or *Emx1+* cells to specifically eliminate D2Rs from developing cortical GABAergic and glutamatergic neurons. We first conducted two experiments to validate this approach. First, confirmed patterns of Cre-induced recombination by inter-crossing ROSA reporter mice to *Nkx2.1*-Cre and *Emx1*-Cre mice followed by immunostaining for cell-specific markers. These mice express tdTomato fluorescence following Cre-mediated recombination. Consistent with an interneuron identity, *Nkx2.1*-Cre-induced recombination was present in scattered cerebral cortical cells throughout the ACC (Figures 1B,F). Interneurons immunoreactive for PV (Figure 1C) and SST (Figure 1G) in fact expressed tdTomato fluorescence (Figures 1D,H). In contrast, *Emx1*-Cre-induced recombination was present in a dense plexus of cells and processes throughout the ACC (Figures 1J,N; and other cerebral cortical regions, data not shown), but interneurons immunoreactive for PV (Figure 1K) and SST (Figure 1O) were almost never positive for tdTomato (Figures 1L,P), consistent with the identity of the recombined cells being glutamatergic pyramidal neurons rather than GABAergic interneurons. These cells were instead typically immunoreactive for protein phosphatase 1 regulatory subunit 1B (also known as DARPP-32, data not shown), a marker for pyramidal cells in dopaminoceptive cortical regions. DAPI-counterstained fields are displayed as (Figures 1A,E,I,M). No effects of sex were noted. Cells in other regions of the CNS were labeled as well (Supplementary Figures 1, 2). Low-power photomicrographs again show dense labeling throughout the cerebral cortex and hippocampus following *Emx1*-induced recombination, but no cellular labeling outside the telencephalon (Supplemental Figures 1A–C). *Nkx2.1*-induced recombination, in contrast, also labels additional cells in the dorsal and ventral striatum, basal forebrain, hypothalamus, and hippocampus (Supplementary Figures 1D–F, 2). In the striatum, recombined cells are not immunoreactive for DARPP-32, a marker for medium spiny neurons (Supplementary Figures 2D,G,J). Some, but not all, of the recombined neurons instead colocalize the cholinergic marker CHAT (Supplementary Figures 2E,H,K).

**Figure 2:**
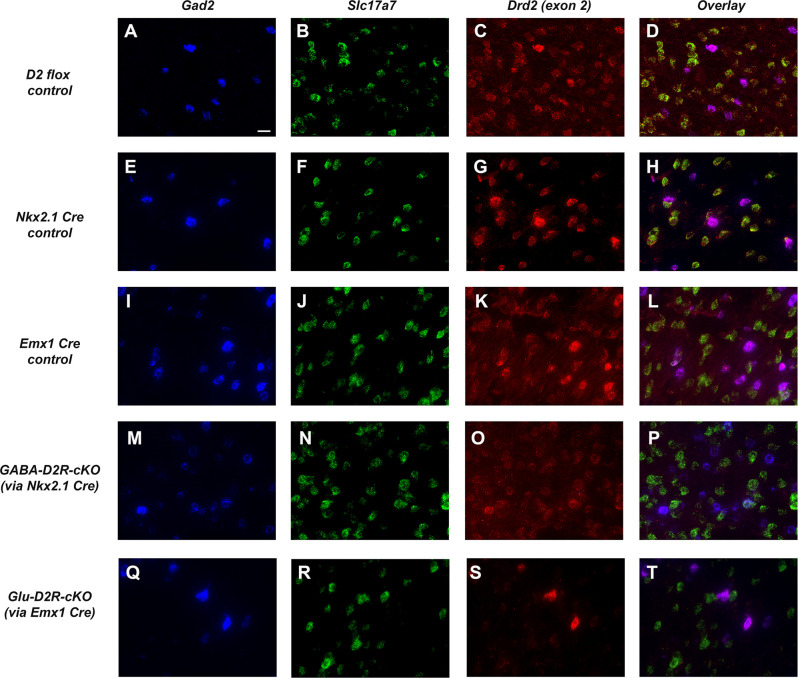
Targeted deletion of D2Rs from ACC GABAergic and glutamatergic neurons in D2R-cKO mouse lines. Representative images using RNAScope^®^-based *in situ* hybridization for *Gad2* (blue, GABAergic neuronal marker; **A,E,I,M,Q**), *Slc17a7* (green, vesicular glutamate transporter; **B,F,J,N,R**), *Drd2* (red,** C,G,K,O,S**), and corresponding multi-color images **(D,H,L,P,T)**. Images were collected with a 60× objective and scale bar = 20 μm. *Drd2* is robustly expressed in both *Gad2*-expressing and *Slc17a7*-expressing cells in this region of the cerebral cortex (ACC) in control mice (panels **A–L**). Note the dramatic reduction in *Drd2* expression in *Gad2*-expressing cells in GABA-D2R-cKO mice (panels** M–P**) but retained expression in *Slc17a7*-expressing cells. Conversely, Glu-D2R-cKO mice retain *Drd2* expression in *Gad2*-expressing neurons (panels **Q–T**) but *Drd2* is not detectable in *Slc17a7*-expressing cells. Images displayed are representative of *N* = 4–6/genotype.

We next used an exon 2-specific *Drd2* probe to label D2R transcripts in order to compare D2R expression (Figures 2C,G,K,O,S) within GABAergic (using *Gad2* probe, Figures 2A,E,I,M,Q) and glutamatergic neurons (using *Slc17a7* probe; Figures 2B,F,J,N,R). *Drd2* was detected in both *Gad2+* and *Slc17a7*+ cells in the ACC of D2 dFlox, *Nkx2.1*-Cre+, and *Emx1*-Cre+ controls (Figures 2D,H,L). As expected based on the patterns of Cre expression, there was a decrease in *Drd2* in *Gad2*+, but not in *Slc17a7*+ cells in the ACC of GABA-D2R-cKO mice (Figures 2O,P). In contrast, Glu-D2R-cKO mice retained expression of *Drd2* in *Gad2+* cells, but it was nearly absent in *Slc17a7*+ neurons (Figures 2S,T). Counts of *Slc17a7+* and *Gad2+* cells that also express *Drd2* were obtained in dFlox controls, Glu-D2R-cKO and GABA-D2R-cKO mice. The percentage of cells double-labeled for *Slc17a*7 *and Drd2* was 80 ± 1.8% in dFlox controls, 5.0 ± 1.3% in Glu-D2R-cKO, and 78.5 ± 4.1% in GABA-D2R-cKO mice (*F*_(2,7)_ = 389.1, *p* < 0.0001). In contrast, the percentage of cells double-labeled for *Gad*2 *and Drd2* was 86 ± 3.2% in dFlox controls, 84.0 ± 5.7% in Glu-D2R-cKO, and 41 ± 6.0% in GABA-D2R-cKO mice (*F*_(2,7)_ = 40.01, *p* = 0.0001). The Cre/loxP system thus resulted in a significant loss of D2R expression in a cell-type-specific (either GABAergic or glutamatergic) manner. Retained *Drd2* in some *Gad2+* neurons is expected since *Nkx2.1+* cells only represent a subset of the total GABAergic neurons in the cerebral cortex. Mice from both cKO lines were physically indistinguishable from controls, were born and survived at expected Mendelian ratios, and did not differ in body weight (data not shown).

### Cortical GABAergic- and Glutamatergic-Specific D2R-cKO Mice Exhibit No Change in Basal Motor Function

**Figure 3:**
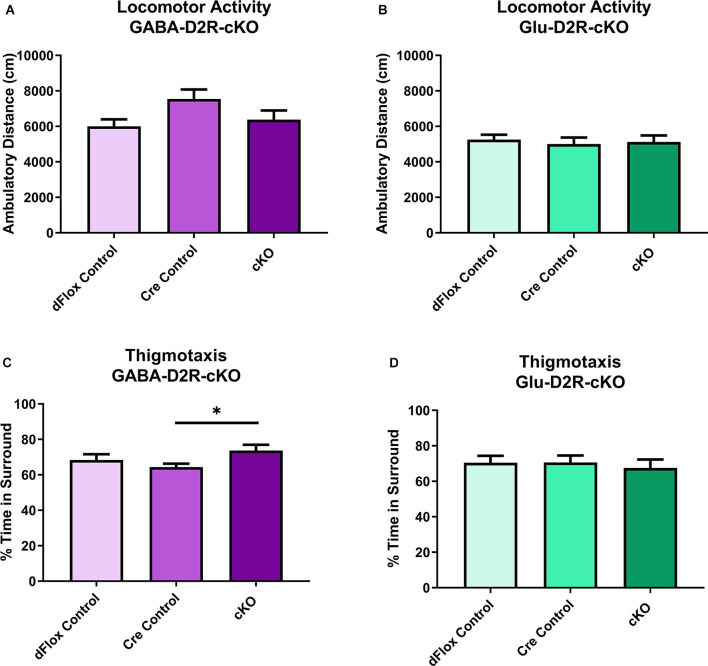
No changes in basal locomotor activity in GABA-D2R-cKO and Glu-D2R-cKO mice. No changes in basal activity levels were apparent in the GABA-D2R-cKO** (A)** or Glu-D2R-cKO **(B,D)** lines, respectively. *Post hoc* analysis did reveal that GABA-D2R-cKO mice did spend more time in the sides and corners of the chamber than the corresponding Cre controls **(C)**. *N* = 10–13/group; *p < 0.05.

To assess locomotor behavior, we tested GABA-D2R-cKO and Glu-D2R-cKO mice in an open field test to measure changes in ambulatory distance. Here we report results collected during a 60-min testing session in the novel open field arena for both D2R-cKO lines; this is the animals’ first exposure to this testing environment. Neither GABA-D2R-cKO (Figure 3A, *F*_(2,34)_ = 2.825, *p* = 0.0733) nor Glu-D2R-cKO (Figure 3B, *F*_(2,29)_ = 0.1405, *p* = 0.8695) mice showed significant differences in distance traveled as compared to its controls. There was a sex-effect with females from all genotypes showing higher measures of distance traveled as compared to males in the GABA-D2R-cKO line (data not shown; *F*_(1,31)_ = 6.520, *p* = 0.0158). In contrast, we did not observe any sex-effect for the Glu-D2R-cKO line (*F*_(1,26)_ = 1.489, *p* = 0.2333). In the GABA-D2R-cKO mice, a significant effect of Genotype (Figure 3C, *F*_(2,31)_ = 3.928, *p* = 0.0302) exhibiting signs of anxiety as demonstrated by thigmotaxis (e.g., preference for dark corners or walls as opposed to spending time in open spaces; Montgomery, 1955). *Post hoc* Bonferroni comparison demonstrated significantly increased time in the surrounding area by the GABA-D2R-cKO mice as compared to Nkx2.1-Cre control mice (*p* = 0.0261) such that cKO mice spent more time against the walls compared to Cre controls (Figure 3C, *F*_(2,31)_ = 3.928, *p* = 0.0302). A modest main effect of Sex was also found in this group, with females exhibiting more anxiety-like behavior (*F*_(1,31)_ = 3.928, *p* = 0.0302). No such effect was found in the Glu-D2R-cKO mice (Figure 3D, *F*_(2,29)_ = 0.1678, *p* = 0.8463).

### Cortical GABAergic-Specific D2R-cKO Mice Exhibit Increased Sensitivity to MK-801

#### MK-801-Induced Locomotor Activity

**Figure 4:**
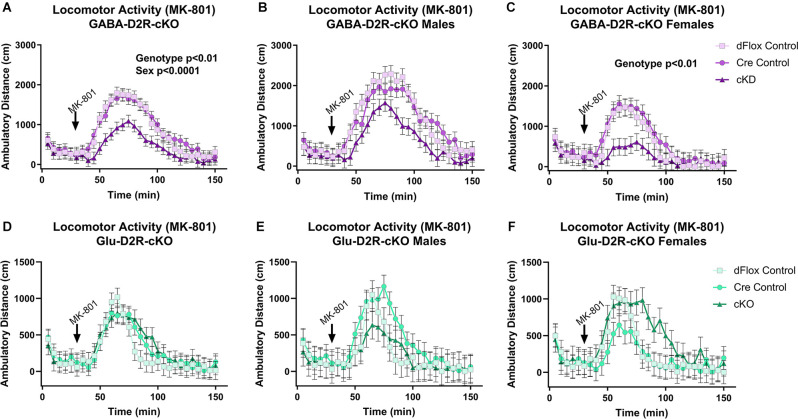
Time course of MK-801-induced locomotor activity in GABA-D2R-cKO and Glu-D2R-cKO mice. MK-801 administration significantly enhanced locomotor activity in the GABAergic **(A–C)** and glutamatergic **(D–F)** D2R-cKO and controls, with GABA-D2R-cKO mice demonstrating a significantly decreased activity compared to controls and a significant difference between males and females of the GABA-D2R-cKO line. Data are shown as collapsed across sex **(A,D)** as well as the activity separately in males **(B,E)** and females **(C,F)**. *N* = 4–7/genotype × sex group.

**Figure 5:**
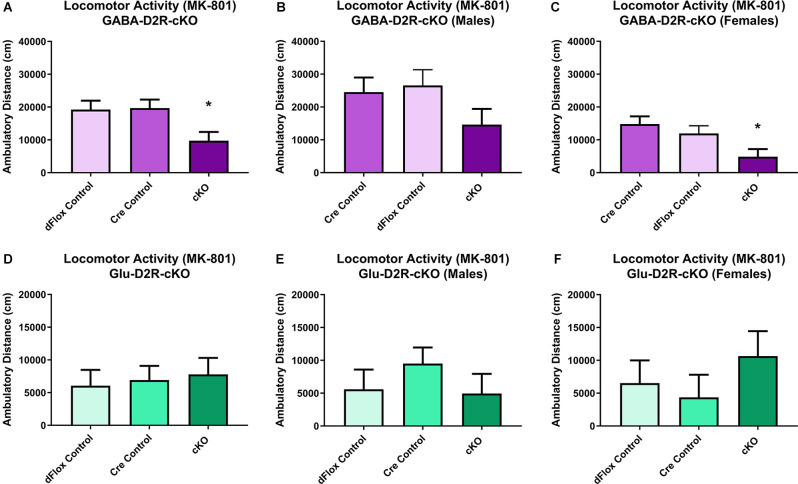
Changes in MK-801-induced locomotor activity in GABA-D2R-cKO mice. Cumulative activity following MK-801 administration in the GABAergic **(A–C)** and glutamatergic **(D–F)** D2R-cKO lines, with significant genotype (*p* < 0.05, Cre, dFlox > KO) and sex (*p* < 0.001) differences only in the GABA-D2R-cKO mice. Data show total activity with males and females combined **(A,D)** and separately within males **(B,E)** and females **(C,F)**. *N* = 4–7/genotype × sex group; *p < 0.05 as compared to either control group by *post hoc* comparison.

Previous behavioral studies have demonstrated that acute administration of MK-801, an NMDA receptor antagonist, induces hyper-locomotion in rodents (Svensson et al., 1991; Diana and Sagratella, 1994). Conversely, administrating antipsychotics that act by blocking D2Rs have been found to reverse behavioral abnormalities elicited in mice with MK-801 administration (Carlsson and Carlsson, 1990; Jentsch and Roth, 1999; Foster et al., 2003). To test whether MK-801 administration could elicit hyperactivity and whether deletion of D2Rs could blunt hyperactive response, we next performed a drug challenge with MK-801, after habituating animals to the chambers over several days. Locomotor activity post-MK-801 administration was analyzed to examine changes in locomotor response to MK-801. MK-801 administration induced a hyperactive phenotype in all mice. However, only the GABA-D2R-cKO mice exhibited profoundly blunted locomotor responses to MK-801 as compared to its controls post-MK-801 administration (Figures 4A–C, 5A–C; *F*_(2,53.5)_ = 6.71, *p* = 0.0025). There was also a main effect of Sex (*F*_(1,53.5)_ = 22.94, *p* < 0.0001), such that females were less sensitive to MK-801’s hyperactive phenotype compared to males; there was no Genotype × Sex interaction (*F*_(2,53.5)_ = 0.45, *p* = 0.6411). *Post hoc* tests confirmed reduced locomotor activity in GABA-D2R-cKO mice as compared to both control genotypes for all mice (Figure 5A), and females only (Figure 5C). Mice from the Glu-D2R-cKO line exhibited hyperactivity following post-MK-801 administration, but there were no differences between the cKOs as compared to control genotypes (Figures 4D–F, 5D–F; *F*_(2,46)_ = 0.21, *p* = 0.8078). Unlike the GABA-D2R-cKO line, there was not an effect of Sex following the MK-801 injection (*F*_(1,46)_ = 0.09, *p* = 0.7686). Based on these responses, only GABA-D2R-cKO mice exhibited a profoundly blunted locomotor response to MK-801, suggesting an increased sensitivity to MK-801.

#### Amphetamine-Induced Locomotor Activity

**Figure 6:**
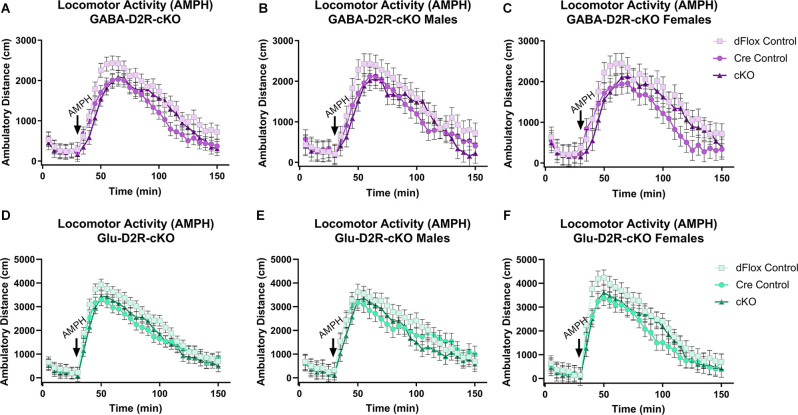
No changes in amphetamine-induced locomotor activity in GABA-D2R-cKO and Glu-D2R-cKO mice. Amphetamine (AMPH) administration induced hyperactivity in all mice, but there were no differences between groups when D2Rs were knocked down in cortical GABAergic interneurons **(A–C)** or glutamatergic neurons **(D–F)**. Data are depicted as collapsed across sex **(A,D)** as well as for males **(B,E)** and females **(C,F)** separately. *N* = 6–7/genotype × sex group.

We then wanted to test whether amphetamine, another psychostimulant known to induce hyperactivity in mice, would differentially alter motor activity in D2R-cKO mice (White et al., 2006). Amphetamine induced a hyperlocomotive response in all genotypes (Figures 6A–C), but there were no significant differences based on Genotype (*F*_(2,51.5)_ = 2.28, *p* = 0.113) or Sex (*F*_(1,51.5)_ = 0.13, *p* = 0.7212). A similar outcome was found within the Glu-D2R-cKO line (Figures 6D–F), with no differences in amphetamine-induced locomotor activity between the genotypes (*F*_(2,53)_ = 1.61, *p* = 0.2088) or sexes (*F*_(1,53)_ = 0.11, *p* = 0.745). Deletion of the D2R from cortical GABAergic or glutamatergic neurons did not alter amphetamine-induced locomotor activity.

### Cortical GABAergic- and Glutamatergic-Specific D2R-cKO Mice Exhibit No Change in Anxiety and Depression-Related Behavior

**Figure 7:**
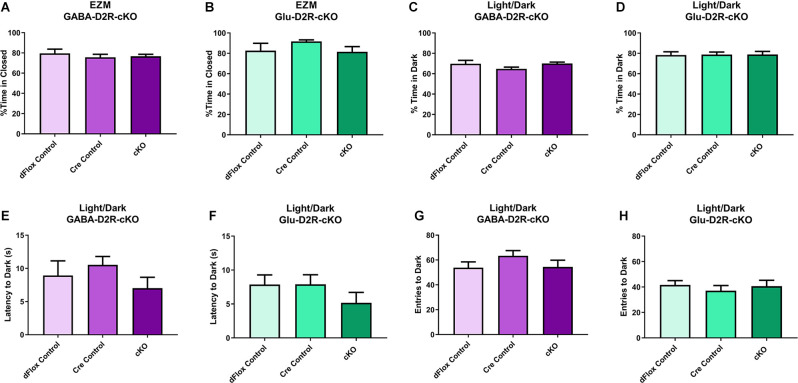
No changes in anxiety-like behavior in GABA-D2R-cKO and Glu-D2R-cKO mice. GABA-D2R-cKO **(A,C,E,G)** and Glu-D2R-cKO **(B,D,F,H)** mice did not differ in anxiety measurements, as measured by the EZM **(A,B)** and light/dark test **(C–H)**. *N* = 9–13/group.

We next tested the D2R-cKO mice using two commonly applied tests used to measure anxiety-related behaviors; the elevated zero maze (EZM) and the light/dark tests. On the EZM, we measured both the percent of time spent in the closed and open areas of the maze. Neither GABA-D2R-cKO (Figure 7A, *F*_(2,28)_ = 0.2258, *p* = 0.7993) nor Glu-D2R-cKO (Figure 7B; *F*_(2,24)_ = 1.734, *p* = 0.1980) mice exhibited significant differences in percent time spent in closed arm as compared to its controls. There was no sex effect in GABA-D2R-cKO (*F*_(1,30)_ = 0.9316, *p* = 0.3422) and Glu-D2R-cKO (*F*_(1,24)_ = 1.197, *p* = 0.2848) mice. In the light/dark test, we report the percent of time spent in the dark, latency to the first entry to the dark, and the number of transitions between the light and dark sides. Similar to EZM, in the light/dark test, neither GABA-D2R-cKO (Figure 7C, *F*_(2,31)_ = 1.392, *p* = 0.2637) nor Glu-D2R-cKO (Figure 7D, *F*_(2,26)_ = 0.0090, *p* = 0.9910) mice showed significant genotype-dependent differences. There was no sex effect in the GABA-D2R-cKO (*F*_(1,31)_ = 0.1875, *p* = 0.8920) nor the Glu-D2R-cKO lines (*F*_(1,26)_ = 3.742, *p* = 0.0640; data not shown); there were no Genotype × Sex interactions. Similarly, there were no genotype-dependent differences in latency to enter the dark compartment (GABA-D2R: Figure 7E, *F*_(2,31)_ = 1.642, *p* = 0.2100; Glu-D2R: Figure 7F, *F*_(2,26)_ = 1.069, *p* = 0.3579) or in the total number of entries (transitions) into the dark (GABA-D2R: Figure 7G, *F*_(2,31)_ = 0.9390, *p* = 0.4019; Glu-D2R: Figure 7H, *F*_(2,26)_ = 0.4392, *p* = 0.6493).

**Figure 8:**
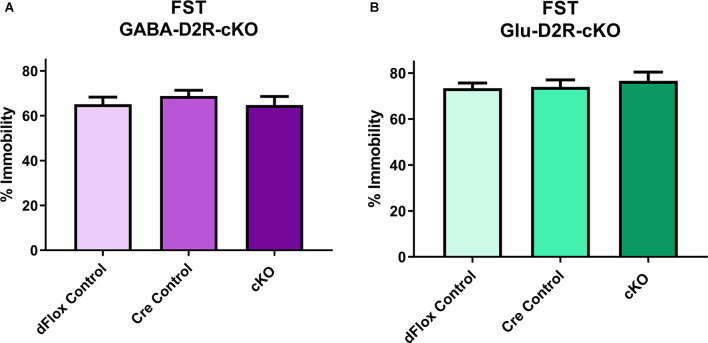
No changes in depression-related behavior in GABA-D2R-cKO and Glu-D2R-cKO mice. No changes in immobility were detected in either the GABA-D2R-cKO **(A)** or the Glu-D2R-cKO **(B)** lines when tested in the FST. Data depict the percent of time spent immobile in the final 4 min of the test. *N* = 11–12/group.

To assess possible changes in depression-related behavior we used the FST. In the FST, neither the full 6-min testing session (*F*_(2,31)_ = 0.91, *p* = 0.41) nor analysis of the last 4-min of the session (Figure 8A, *F*_(2,28)_ = 0.5152, *p* = 0.6030) revealed significant differences in the percent of immobility between GABA-D2R-cKO mice as compared to its controls. Similarly, neither the full 6-min testing session (*F*_(2,30)_ = 0.3872, *p* = 0.6823) nor the last 4-min testing session (Figure 8B, *F*_(2,30)_ = 0.01382, *p* = 0.9863) showed significant differences in the percent of immobility between Glu-D2R-cKO mice as compared to its controls. There were no significant effects of sex for either the GABA-D2R-cKO (*F*_(1,28)_ = 0.4635, *p* = 0.5051) or Glu-D2R-cKO (*F*_(1,30)_ = 1.801, *p* = 0.1897) mice for the full 6-min testing session as well as for the GABA-D2R-cKO (*F*_(1,28)_ = 0.2055, *p* = 0.6538) and Glu-D2R-cKO (*F*_(1,30)_ = 3.084, *p* = 0.0893) mice for the last 4-min testing session.

### Cortical GABAergic- and Glutamatergic-Specific D2R-cKO Mice Exhibit No Change in Tests of Short-Term and Working Memory

**Figure 9:**
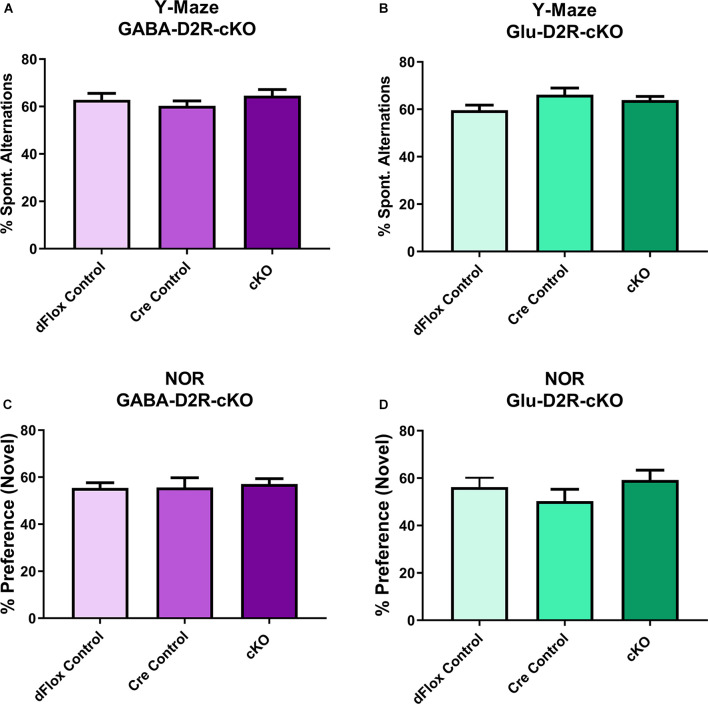
No changes in short-term and working memory in either GABA-D2R-cKO or Glu-D2R-cKO mice. Performance in neither the Y-maze **(A,B)** nor the NOR test **(C,D)** resulted in changes in the GABA-D2R-cKO **(A,C)** or Glu-D2R-cKO mice. *N* = 8–13/group.

To assess short-term and working memory, we used the Y-maze and NOR tests, since both tests are commonly used to assess changes in short-term and working memory tasks (Miedel et al., 2017). For Y-maze, neither GABA-D2R-cKO (Figure 9A, *F*_(2,31)_ = 0.2523, *p* = 0.7786) nor Glu-D2R-cKO (Figure 9B, *F*_(2,26)_ = 1.871, *p* = 0.1740) mice exhibited significant differences in percent spontaneous alternations as compared to its controls, where chance performance was defined as 22.2%. There was no sex-effect in either GABA-D2R-cKO (*F*_(1,31)_ = 1.559, *p* = 0.2212) and Glu-D2R-cKO (*F*_(1,26)_ = 0.04829, *p* = 0.8278) mice. Similar to the Y-maze, in the NOR test, neither GABA-D2R-cKO (Figure 9C, *F*_(2,31)_ = 0.6277, *p* = 0.5404) nor Glu-D2R-cKO (Figure 9D, *F*_(2,26)_ = 0.3569, *p* = 0.7032) mice exhibited significant differences in % preference to the novel object as compared to its controls. Chance performance value for novel/familiar ratio was defined as a value of 50%; preference scores above this value indicate novel object preference and below this index, there is no preference for the novel object, as previously described (Hammond et al., 2004). We note that in our experiments, the control mice displayed only a very modest preference for the novel object, but once again, there were no differences between control and cKO mice in either line. There were also no sex-effects in both GABA-D2R-cKO (*F*_(1,31)_ = 0.08683, *p* = 0.7702) and Glu-D2R-cKO (*F*_(1,26)_ = 0.9362, *p* = 0.3422 mice. Overall, results from Y-maze and NOR test suggest no change in short-term and working memory in both D2R-cKO mice.

### Cortical GABAergic-Specific, but Not Glutamatergic-Specific, D2R-cKO Mice Exhibit Changes in Motor Coordination

**Figure 10:**
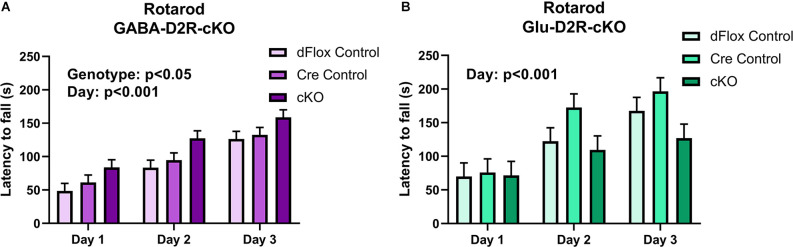
Changes in motor coordination in GABA-D2R-cKO mice. While performance on the rotarod improved over time in both the GABA-D2R-cKO **(A)** and the Glu-D2R-cKO **(B)** lines, only mice with decreased D2Rs in cortical interneurons performed significantly better than its respective controls. *N* = 8/group for Glu-D2R-cKO mice and 25–26/ for GABA-D2R-cKO (several cohorts were run to confirm the effects).

Despite no changes in basal motor function in both D2R-cKO mice, we wanted to further test for changes in motor coordination and motor learning *via* the rotarod test. Within the GABA-Drd2-cKO cohort, mice from all genotypes showed improvement in motor learning over time without any interaction between Day of training and Genotype. There was a significant effect of Genotype for GABA-Drd2-cKO (Figures 10A,B; *F*_(2,67.8)_ = 4.18, *p* = 0.0195) as compared to both controls, showing increased latency to fall for all three days of training. There was also a significant effect on days of trial (*F*_(2,96.9)_ = 62.77, *p* < 0.001), with a gradual increase in latency to fall from Day 1 of training to Day 3 of training. There was no significant main effect of Sex (*F*_(1,67.8)_ = 0, *p* = 0.9543), nor were there significant interactions of any sort. These results suggest that GABA-D2R-cKO mice exhibit enhanced motor coordination as assessed by increased latency to fall even from Day 1 of training up to Day 3 of training as compared to its controls. For the Glu-D2R-cKO mice, there was no significant effect on genotype (Figure 10B; *F*_(2,18)_ = 1.87, *p* = 0.1827), although there was a significant effect on days of trial (*F*_(2,27.1)_ = 23.54, *p* < 0.001), with a gradual increase in latency to fall from Day 1 of training to Day 3 of training. No main effect of Sex was found (*F*_(1,18)_ = 0.18, *p* = 0.6801), nor was there a significant interaction of genotype and day (*F*_(4,28.7)_ = 1.95; *p* = 0.1297) in the Glu-D2R-cKO line. Visual inspection of day 3 on Figure 10B suggests a potential decrease in performance in Glu-D2R-cKO mice, but this was not statistically significant. Overall, our results suggest that we observed enhancement in motor coordination in GABA-D2R-cKO mice and no changes in motor coordination in Glu-D2R-cKO mice (although we note a possible trend to decreased motor learning).

### Only Cortical GABAergic-Specific D2R-cKO Mice Exhibit Deficits in Spatial and Reversal Learning

**Figure 11:**
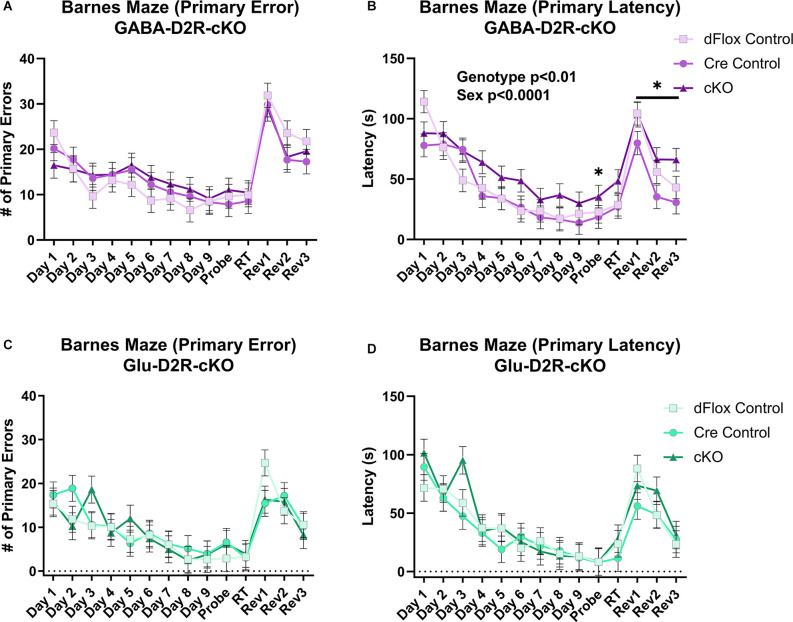
Deficits in spatial learning memory in GABA-D2R-cKO mice. Only the GABA-D2R-cKO mice **(A,B)** showed a deficit in function compared to controls in primary latency **(B,D)** but not the primary error **(A,C)**. No such changes were observed in Glu-D2R-cKO mice **(C,D)**. *N* = 7–8/group for Glu-D2R-cKO mice and 18–19/ for GABA-D2R-cKO (several cohorts run to confirm the effects). *p < 0.05 as compared to controls by *post hoc* comparison.

To test for spatial and reversal learning, we used the Barnes maze test, which consisted of acquisition training for the first nine consecutive days, followed by a probe test, re-training, and reversal learning for three consecutive days. Overall, there was no effect on primary error based on Genotype (*F*_(2,44.5)_ = 0.1, *p* = 0.9021) or Sex (*F*_(1,44.5)_ = 0.02, *p* = 0.8873) in the GABA-D2R-cKO line (Figure 11A). However, there was a main effect of Genotype (*F*_(2,51.8)_ = 7.7, *p* = 0.0012) and Sex (*F*_(1,51.8)_ = 15.91, *p* = 0.0002) on primary latency. In this regard, cKO mice took significantly longer to escape than controls (Figure 11B). There was not a corresponding significant Genotype × Sex interaction (*F*_(2,51.8)_ = 0.71, *p* = 0.498) on primary latency in the GABA-D2R-cKO line. On the contrary, there was no overall differences in primary error (Figure 11C) and primary latency (Figure 11D) due to Genotype [error: (*F*_(2,17.5)_ = 0.08, *p* = 0.9229); latency: (*F*_(2,17.2)_ = 0.8, *p* = 0.4653)] or Sex [error: (*F*_(1,17.5)_ = 0.98, *p* = 0.3362); latency: (*F*_(1,17.2)_ = 1.82, *p* = 0.1946)] in the Glu-D2R-cKO line. Below, we show results when phases were analyzed separately.

#### Acquisition Training

Throughout acquisition training (Days 1–9, Figure 11), there were no significant differences between GABA-D2R-cKO mice and its controls in primary error due Genotype (*F*_(2,47.3)_ = 0.56, *p* = 0.5767) or Sex (*F*_(1,47.3)_ = 0.62, *p* = 0.4351). However, GABA-D2R-cKO mice found the escape box somewhat slower than both controls as measured by primary latency due to Genotype (*F*_(2,46.5)_ = 2.96, *p* = 0.0619), albeit not significantly. Neither Sex (*F*_(1,46.5)_ = 5.18, *p* = 0.0275) nor a Genotype × Sex interaction (*F*_(2,46.5)_ = 0.25, *p* = 0.7827) was evident. For the Glu-D2R-cKO line, there were no changes in primary error due to Genotype (*F*_(2,17.7)_ = 0.27, *p* = 0.7649) or Sex (*F*_(1,17.7)_ = 0.93, *p* = 0.3482) or in primary latency based on Genotype (*F*_(2,17.3)_ = 0.43, *p* = 0.6547) or Sex (*F*_(1,17.3)_ = 0.59, *p* = 0.4537). In terms of search strategy, all mice, regardless of genotype or mouse line, utilized the more disorganized random search strategy similarly in the initial training, before moving on to the more complex serial and spatial strategies over time (data not shown). Acquisition training results suggest that only GABA-D2R-cKO mice exhibited minor spatial learning and memory deficits as exhibited by primary latency.

#### Probe Test

During the probe test following acquisition training (Probe, Figure 11), GABA-D2R-cKO mice found the escape box much slower than both controls as measured by primary latency (*F*_(2,49)_ = 3.54, *p* = 0.0367) but not primary error (*F*_(2,49)_ = 0.48, *p* = 0.6225). The performance between males and females did not differ (error: *F*_(1,49)_ = 0.55, *p* = 0.4599; latency: *F*_(1,49)_ = 0.86, *p* = 0.3583). In contrast, there were no significant differences in the Glu-D2R-cKO mouse line due to Genotype or Sex in primary error (Genotype: *F*_(2,17)_ = 0.41, *p* = 0.6682; Sex: *F*_(1,17)_ = 2.63, *p* = 0.1231) or primary latency (Genotype: *F*_(2,17)_ = 0.01, *p* = 0.995; Sex: *F*_(1,17)_ = 0.8, *p* = 0.3823). Probe test results suggest that, unlike other mice, only GABA-D2R-cKO mice could not retain spatial memory learned throughout acquisition training, suggesting a spatial learning deficit in cortical GABAergic-specific D2R-cKO mice.

#### Reversal Training

During reversal training (Rev 1–3, Figure 11), GABA-D2R-cKO mice did not show significant differences between its controls in primary error (*F*_(2,45.3)_ = 0.71, *p* = 0.4949) but did show significant differences in primary latency (*F*_(2,38.8)_ = 3.88, *p* = 0.0291) based on Genotype, with the cKOs taking significantly more time in finding the escape hole compared to Cre controls. There was also a significant effect of Sex in primary latency (*F*_(1,38.8)_ = 11.13, *p* = 0.0019), with males having longer latencies than females. No Genotype × Sex interaction (*F*_(2,38.8)_ = 0.25) was found in primary latency although there was a main effect of Day (*F*_(2,63.5)_ = 18.49, *p* < 0.0001), with latencies decreasing on each subsequent day. In regards to primary error, males committed more primary errors than females (Sex: *F*_(1,45.3)_ = 4.04, *p* = 0.0504), albeit non-significantly, and there was a significant effect of Day (*F*_(2,68.7)_ = 13.86, *p* < 0.0001), with mice again demonstrating improvement over subsequent sessions.

In the Glu-D2R-cKO mouse line, there was no change in primary error based on Genotype (*F*_(2,17.8)_ = 0.39, *p* = 0.681) or Sex (*F*_(1,17.8)_ = 0.01, *p* = 0.9234), although fewer errors were committed over each reversal testing session (Day: (*F*_(2,23.7)_ = 8.27, *p* = 0.0019). Neither Genotype (*F*_(2,18.7)_ = 0.78, *p* = 0.4737) nor Sex (*F*_(1,18.7)_ = 3.96, *p* = 0.0613) affected primary latency in the reversal trials, although again latencies decreased throughout the sessions (Day: *F*_(2,26.9)_ = 7.95, *p* = 0.0019). Overall, acquisition training, probe test, and reversal learning results suggest that only GABA-D2R-cKO mice exhibited spatial learning and memory deficits as indicated through increased latencies to find the escape hole, without changes in behavioral strategy or performance as indicated by no change in errors.

### Neither Cortical GABAergic nor Glutamatergic Neuronal Deletion of D2R Altered GAD67 Immunoreactivity in the ACC

**Figure 12:**
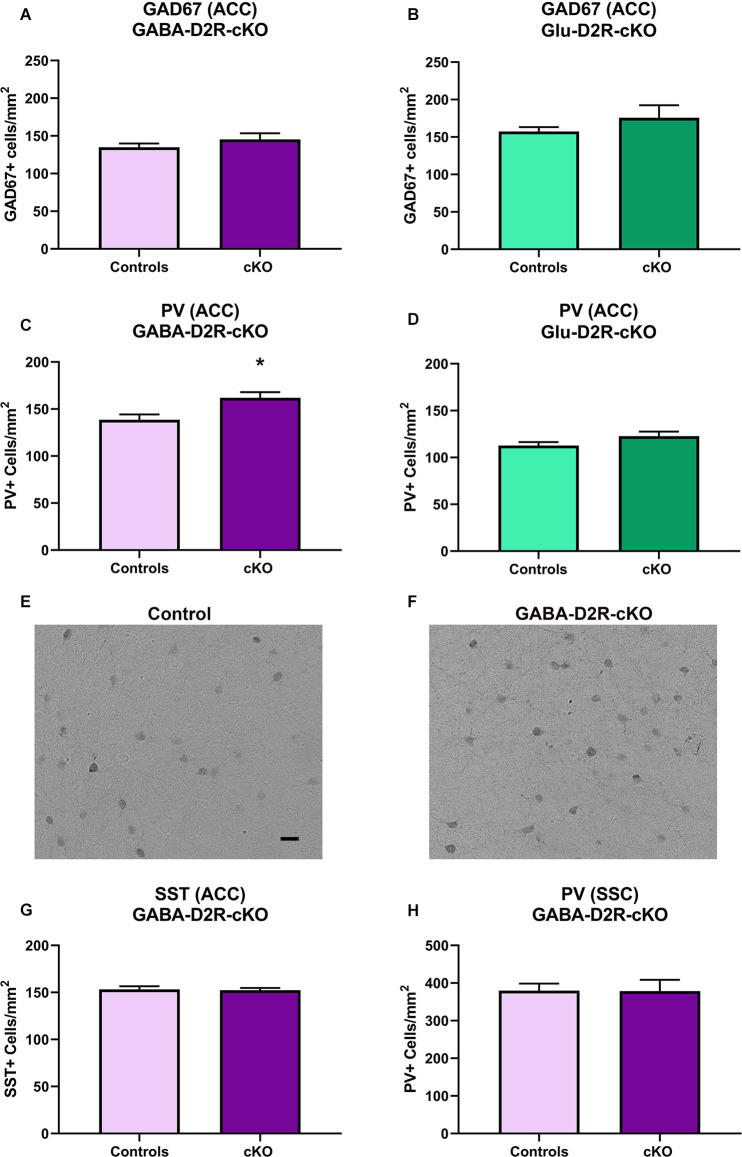
Increased PV+ interneurons in GABA-D2R-cKO mice in the absence of changes in other markers or brain regions. The number of cortical GAD67+ neurons did not differ in either GABA-D2R-cKO **(A)** or Glu-D2R-cKO **(B)** mice. On the contrary, PV+ interneurons within the ACC were significantly elevated in GABA-D2R-cKO **(C,F)** compared to controls **(E)**, but no change was apparent in Glu-D2R-cKO **(D)** mice. This effect was specific to this marker within this region, as neither SST numbers **(G)** nor PV+ interneurons within the DA-poor SSC region **(H)** changed. *p < 0.05 as compared to control by *post hoc* comparison.

Previously, our lab has reported increased numbers of PV interneurons and overall GABAergic interneurons in the ACC from adult mice with marked differences as early as P14 following the global deletion of D2Rs in all cells throughout development (Graham et al., 2015b). Studies have also consistently reported reduced GAD67 levels in schizophrenic patient brains as well as in other subtypes of GABAergic interneurons, including PV and SST, which is believed to contribute to impaired inhibitory inputs onto excitatory pyramidal cells (Gonzalez-Burgos et al., 2010). To further assess neuroanatomical changes related to various neuropsychiatric disorders, we assessed changes in GAD67 immunoreactivity in the ACC. There were no significant differences in GAD67+ cell density in GABA-D2R-cKO mice as compared to its controls in the ACC (Figure 12A, *t*_(22)_ = 1.139, *p* = 0.2668). Similarly, there were no significant differences in GAD67+ cell density in Glu-D2R-cKO mice as compared to the combined controls in the ACC (Figure 12B, *t*_(29)_ = 1.246, *p* = 0.2227). Our results suggest that there is no change in the number of cortical GAD67+ neurons in the ACC following the deletion of D2Rs in either GABAergic or glutamatergic neurons.

### Cortical GABAergic-Specific D2R-cKO Mice Exhibit Increased PV Immunoreactivity in the ACC

Fast-spiking PV interneurons are crucial in the regulation of synchronized neuronal activity, with dysregulation of PV interneurons linked to the pathophysiology of schizophrenia, bipolar illness, and substance abuse (Rotaru et al., 2012). Based on our previous findings that the constitutive loss of the D2R increased cortical PV+ neuron numbers (Graham et al., 2015b), we hypothesized that there would be changes in this subtype of GABAergic interneurons. As expected, we did find a significant increase in PV+ neurons in the ACC of GABA-D2R-cKO mice compared to its controls (Figures 12C,E,F, *t*_(24)_ = 2.486, *p* = 0.0203). On the contrary, there were no significant differences between PV+ cell numbers in the ACC of the Glu-D2R-cKO mice and its controls (Figure 12D, *t*_(29)_ = 1.598, *p* = 0.1209). Although quantitative cell counts were not obtained in individual cortical layers, the increase in PV+ cells appears to be homogeneous across the depth of the cortex and therefore not lamina-specific. This change in GABAergic interneurons is specific to both the GABAergic interneuron subtype as well as the brain region, as there were no differences in the number of SST+ neurons in the ACC (Figure 12G; *t*_(21)_ = 0.1408, *p* = 0.8894), despite the presence of recombination in SST+ neurons (Figures 1E–H), nor the number of PV+ neurons between genotypes in the DA-poor SSC (Figure 12H; *t*_(34)_ = 0.03426, *p* = 0.9729), similar to our previous findings (Graham et al., 2015b). Our results suggest that there is an increased PV interneuron density specifically in the ACC upon deleting D2Rs from cortical GABAergic interneurons from the Nkx2.1 lineage.

## Discussion

### Cell-Type-Specific Deletion of D2Rs in Cerebral Cortical GABAergic or Glutamatergic Neurons

Cerebral cortical neurons are broadly classified into glutamatergic excitatory projection neurons and GABAergic inhibitory neurons. Pyramidal neurons account for ~80% of all neurons in the cerebral cortex and provide output to other cortical and subcortical regions; interneurons represent the remaining ~20% of neurons and form local synaptic connections to shape the cortical network activity patterns (Han and Sestan, 2013). Despite this bias in absolute numbers, the interneurons may ultimately provide the more prominent influence on the balance of excitation and inhibition, and determine functional activation patterns, especially with respect to upper-level associative functions, such as emotion regulation, cognition, and executive function. Disruption of a number of important neurodevelopmental influences, including DA homeostasis, has been implicated in a variety of neuropsychiatric disorders, including attention deficit hyperactive disorder, schizophrenia, and mood disorders. Inappropriate expression or activity of GABAergic interneurons accompany and may underlie these disorders. We previously discovered persistent changes in the number, morphology, and function of cerebral cortical interneurons within the ACC of DA D1R and D2R knockout mice (Stanwood et al., 2005; Graham et al., 2015b).

Cerebral cortical functions of D2Rs are of considerable interest. DAergic transmission in the medial frontal cortex and other brain regions contributes to the regulation of fear, anxiety, attention, and working memory. Moreover, genes related to DA and especially D2R functional pathways are associated with multiple neuropsychiatric diseases, and D2Rs are a target of multiple medications to treat mood and thought disorders (Li et al., 2016; Ashok et al., 2017; Pergola et al., 2017; Faraone, 2018; Alexopoulos, 2019; Selvaggi et al., 2019). Although early studies suggested that D2Rs are expressed modestly in the cerebral cortex (<20% of neurons, including very few interneurons), studies using more sensitive detection have demonstrated D2R expression in at least a third of cortical interneurons (dependent on subclass), and in up to 50% of glutamatergic excitatory neurons (Santana and Artigas, 2017; Khlghatyan et al., 2019). D2Rs contribute to cognitive flexibility by engaging a more flexible state of working memory networks (Durstewitz and Seamans, 2008; Stokes et al., 2013; Ott and Nieder, 2017). For example, D2R stimulation in the prefrontal cortex of monkeys engaged in a cognitive task increases working memory encoding at both single neuron and population levels (Ott and Nieder, 2017). The resting and active properties of PV+ GABAergic interneurons are targets of D2R modulation (Cousineau et al., 2020). Moreover, a schizophrenia-related genetic deletion reduces the ability of D2Rs to gate the inhibition of excitatory neurons through PV+ inhibitory neurons in the cerebral cortex (Choi et al., 2018), providing a neural mechanism linking D2R-mediated function specifically in GABAergic interneurons to the pathophysiology of psychosis. The vital role of neurodevelopmental adaptations/maladaptations in the generation of psychosis-related pathophysiology has been recently demonstrated by the ability of D2R blockade during adolescence preventing the onset of schizophrenia in a genetic animal model (Mukherjee et al., 2019).

To target deletion of D2Rs from each of the major cerebral cortical cell types, we generated two cell-type-specific D2R-cKO mice *via* Cre/loxP system by consecutively breeding exon 2-specific *Drd2*^flox/flox^ with either *Nkx2.1*-Cre to target deletion of D2Rs in cerebral cortical GABAergic interneurons or *Emx1*-Cre to target deletion of D2Rs in telencephalic glutamatergic neurons. For cortical GABAergic interneurons, we used the homeobox transcription factor, *Nkx2.1*, which is specifically expressed within cells of the developing medial ganglionic eminence (MGE) and controls the regional identity of MGE progenitors by influencing the cell-fate specification of these MGE-derived interneurons in a temporarily defined manner (Sussel et al., 1999). From the ventricular and sub-ventricular progenitor zones, many *Nkx2.1*+ cells then undergo migration to the cerebral cortex to become GABAergic interneurons, specific for the PV and SST subclasses (Sussel et al., 1999; Xu et al., 2004; Liodis et al., 2007; Du et al., 2008; Nobrega-Pereira et al., 2008). We note, however, that Nkx2.1 is also an important transcription factor in the specification of interneurons to other telencephalic regions, including the hippocampus (Chittajallu et al., 2013; Asgarian et al., 2019), and for the differentiation of specific cholinergic neurons in the basal forebrain (Magno et al., 2017). Previous studies have demonstrated that loss of *Nkx2.1* (E9.5-E12.5) causes a significant cell-autonomous decrease in MGE-derived subtypes (PV and SST interneurons; Butt et al., 2008). By targeting deletion of D2Rs from *Nkx2.1*+ cells during development, we aimed to delete D2Rs from a subset of cortical GABAergic interneurons in our GABA-D2R-cKO mice. Our crosses with a ROSA reporter line and ISH analyses verified the fidelity with which cell-specific knockdown/knockout of *Drd2* was achieved. We used another homeobox transcription factor, *Emx1*, to complementarily target deletion of D2Rs from cortical pyramidal neurons (Gulisano et al., 1996; Chan et al., 2001). Based on previous studies, *Emx1* expression is restricted primarily to cortical subdivisions of the telencephalon and is known to characterize most excitatory cortical neurons during proliferation, differentiation, migration, and postnatal development and starts being expressed as early as E10 (Simeone et al., 1992; Briata et al., 1996; Gulisano et al., 1996; Puelles et al., 2000). In support of this, studies have demonstrated that radial glia, Cajal-Retzius cells, and glutamatergic neurons of most pallial structures, but not GABAergic interneurons, originate from an *Emx1*-expressing lineage (Chan et al., 2001; Gorski et al., 2002) and migrate radially to their final location (Tan et al., 1998; Hammond et al., 2001).

### Behavioral and Cellular Alterations in GABA-D2R-cKO Mice

Our data indicate that loss of D2Rs from glutamatergic neurons within the frontal cortex (Glu-D2R-cKO line) had no significant effects on adult behavior patterns, despite a wide-ranging set of behavior modalities and measurements. Assays included assessment of motor behavior and learning (basal and pharmacological challenges in open field, rotarod, entries on multiple mazes), anxiety and depression-related behavior (thigmotaxis in open field, light/dark chamber, EZM, forced-swim test), and memory (spontaneous alternation in a Y maze, NOR, Barnes maze).

On the contrary, loss of D2R from GABAergic interneurons (GABA-D2R-cKO line) altered specific aspects of behavior and cognition. GABA-D2R-cKO mice were significantly more coordinated than their controls, as assessed by the rotarod. The circuit mechanisms underlying this difference in the GABA-D2R-cKO mice are unclear, but both dopamine and corticostriatal inputs are known to modulate action initiation, motor responses and plasticity, and habit learning (Costa, 2007; Klaus et al., 2019). Moreover, administration of the NMDA receptor antagonist MK-801 significantly increased locomotor activity in GABA-D2R-cKO mice and associated controls, with females being less sensitive to MK-801’s locomotor-stimulating effects compared to males. Others have previously noted that males and females respond differently to MK-801: male mice are more hyperlocomotive following MK-801 administration compared to females (van den Buuse et al., 2017), but the opposite effect has been documented in rats (Blanchard et al., 1992; Hönack and Löscher, 1993; McDougall et al., 2020); species differences and dose appear to modulate these differential outcomes. Furthermore, in addition to the overall blunted hyperactivity following MK-801, the response by GABA-D2R-cKO females was blunted even more so relative to their respective controls. D2R activation depresses NMDA-receptor mediated responses *via* a receptor tyrosine kinase (Kotecha et al., 2002). Further, studies attempting to examine the underlying circuitry of schizophrenia have demonstrated that DA, glutamate, and GABA circuitries are intertwined within the frontal cortex. Specifically, MK-801 increases cortical DA release, which in turn decreases GABAergic inhibition by activation of D2 autoreceptors (López-Gil et al., 2009). The exploitation of the circuitry is the reason D2R antagonists are often used to treat schizophrenic psychosis. Moreover, there is some evidence that the number of DAergic neurons, dopamine receptors, and NMDA receptors differs between the sexes, a dichotomy that may occur during development (Beyer et al., 1991; Andersen and Teicher, 2000; Wang et al., 2015; Orendain-Jaime et al., 2016). Inactivation of *Drd2* in striatal medium-spiny neurons also has been demonstrated to influence locomotor responsiveness to MK-801 (Kharkwal et al., 2016), and cKO of Drd2 from Wolfram syndrome 1+ cells accentuates the locomotor effects of amphetamine, but not MK-801 (Puighermanal et al., 2020). Given that many neuropsychiatric disorders with a developmental etiology, such as schizophrenia, depression, and attention-deficit hyperactivity disorder, exploit these circuits and present preferentially in one sex over the other, understanding these changes is of great importance. However, these data are in contrast to what was observed in the MK-801-treated Glu-D2R-cKO mice, whereby no sex or genotype differences were found, indicating that the D2Rs expressed within GABAergic interneurons are the critical subpopulation underlying these changes. Additionally, loss of D2R in either cell population neither altered basal nor amphetamine-induced locomotor activity. Importantly, the GABA-D2R-cKO mice also likely exhibit loss of D2 receptors from cholinergic interneurons in the striatum and nucleus accumbens (Magno et al., 2017), and loss of those receptors may contribute to the observed phenotypes given the roles for D2R regulation of striatal cholinergic functions (Gallo et al., 2021; Simpson et al., 2021).

GABA-D2R-cKO mice also demonstrated mild cognitive impairments in the Barnes maze which cannot be attributed to anxiety or ambulatory deficits, per EZM, light/dark, and basal locomotor activity results. D2Rs within the prefrontal cortex are essential for appropriate cognitive function (Arnsten et al., 1995; Wang et al., 2004; Floresco, 2013; Papenberg et al., 2020), and these data indicate that D2Rs located on GABAergic interneurons are essential to these functions. These observations are further supported by a recent study in which D2Rs were eliminated from PV-expressing GABAergic neurons throughout the brain (Tomasella et al., 2018). Those conditional mutants exhibited changes in behavior on an elevated plus maze, marble burying assay, Y maze and NOR, as well as reductions in pyramidal neuron dendritic spine density and hippocampal electrophysiological properties (Tomasella et al., 2018). Both amphetamine-induced and MK-801-induced locomotor activity was modestly increased in those mutants, effects that were ameliorated by the administration of antipsychotic drugs. Of note, PV expression initiates several weeks later than Nkx2.1, specifically during the 2nd postnatal week in the cerebral cortex, after neurogenesis and migration have concluded. Furthermore, PV is expressed in other brain regions and cell types beyond cortical and hippocampal interneurons, including neurons in the cerebellum, hypothalamus, midbrain, and select thalamic subregions (Celio and Heizmann, 1981; Celio, 1986, 1990; Del Río et al., 1994).

The relatively modest effects of D2R-KO in this study were somewhat unexpected. In addition to the potent effects of D2R modulation on cerebral cortical structure and function described above, we have previously shown that constitutive loss of the D2R results in a robust antidepressant-like phenotype. We also demonstrated that the global loss of the D2R increases the number GABAergic interneurons, specifically those containing PV, within the frontal cortex but not in the striatum nor the DA-poor SSC (Graham et al., 2015b). We did note elevated PV+ neuron numbers within the frontal cortex in the current study, but no changes in SST+ neuronal density (although SST+ cerebral cortical cells underwent recombination based on our crosses with the ROSA mice. These studies suggest that these changes to PV+ interneuron number may be at least partially responsible for changes in depression or depressive-like behaviors in humans and rodents, respectively (Shen et al., 2008; Leussis et al., 2012; Kigawa et al., 2014; Graham et al., 2015b; Perlman et al., 2021; Wang et al., 2021). The Rho GTPase-activating protein oligophrenin-1 appears to be a central node in the regulation of interneuron function and mood-related behaviors in the cerebral cortex (Wang et al., 2021); studies are underway in our laboratory to assess changes in oligophrenin-1 expression and function in the D2R-cKO mouse lines.

### Caveats and Next Steps

Our study has several caveats and limitations worth noting. Although we did not observe significant effects of Cre expression in the current study, we created Cre controls using separate breeders rather than creating littermates. This was done to maximize experimental mouse numbers and limit the creation of mice with genotypes unsuitable for experiments. All lines were fully backcrossed, but subtle effects of parental history and rearing environment may have subtly influenced some variables. Cre line specificity is also always a limiting factor—both in terms of developmental timing and cell types. For example, the Nkx2.1-Cre line has also been used to study the modulatory influences of hippocampal interneurons (Akgül and Mcbain, 2020), hippocampal SST+ interneurons are known to contribute to the Barnes maze task (Artinian et al., 2019), and we noted additional brain regions and cell types in which D2Rs may have been inactivated based on ROSA mapping of recombination (see Supplementary Data). Rhinal cortical D2Rs also play important roles in some aspects of learning schedules of trials and reinforcement (Liu et al., 2004), and dysregulation of those receptors may contribute to the observed phenotypes in the current study. The Cre lines we used induced loss of Drd2 quite early in neural development; this almost certainly induces distinct neuro(mal)adaptations as compared to the adult loss. Viral-mediated recombination at different ages and in different brain regions and circuits will be of interest, as will analyses of the ontogeny of altered behaviors and interneurons in the GABA-D2R-cKO mice studied here.

With regard to the altered sensitivity of locomotor responses to MK-801 in GABA-D2R-cKO mice, a dose-response curve and the use of additional NMDA receptor antagonists would demonstrate specificity. Expression analyses and/or electrophysiological characterization of NMDA receptors in cortical interneurons vs. pyramidal cells will also be important. D2R signaling and function are linked to its phosphorylation by G protein receptor kinases and interactions with arrestins, and new reagents are available to probe these differences (Mann et al., 2021). D2Rs expressed by excitatory and inhibitory neurons may signal through distinct protein interactions and/or modifications.

We note also that the locomotor studies using MK-801 and amphetamine were carried out in the two different lines by different experimenters and at different times in the life of this project. Control mice from the GABA-D2R-cKO experiment showed a larger peak response to MK-801 than controls in the Glu-D2R-cKO line. Conversely, amphetamine-induced peak locomotor reactivity appeared higher in the Glu-D2R-cKO line than in the Nkx2.1-Cre counterparts. We do not have an explanation for these differences beyond the typical behavioral study variability observed between distinct cohorts. These findings should be replicated in future experiments. Lastly, we observed retained *Drd2* expression in a good number of cortical *Gad2+* neurons in GABA-D2R-cKO mice. This is expected since *Nkx2.1+* cells only represent a subset of the total GABAergic neurons in the cerebral cortex; however, analysis of additional subpopulations of D2R-expressing interneurons may reveal adaptations in additional features of neuronal circuits and behavior.

### Summary

To our surprise, loss of D2Rs from excitatory neurons in the cerebral cortex (Glu-D2R-cKO) had virtually no long-lasting effects on neurobehavioral function. Use of Nkx2.1-Cre mice to produce GABA-D2R-cKO mice resulted in blunted locomotor responses to the psychotomimetic drug MK-801, improved motor coordination on a rotarod, spatial learning deficits on a Barnes maze, and a significant increase in PV+ interneurons in the ACC. Basal locomotor behavior, anxiety, and depression-related behaviors were unaltered. Our study, therefore, demonstrates that there are unique and distinct roles for D2Rs within excitatory and inhibitory neurons in the regulation of behavior and interneuron development. Location- and/or cell-type biased D2R antagonism may be clinically advantageous for some brain disorders, leading to higher efficacy and reduced side effects. These data also point to modulatory roles for D2Rs in the establishment of behavior, cognition, and cerebral cortical circuits. Further study and direct association of the roles for D2 receptors in GABAergic neurons in different regions of the telencephalon and subcortical cholinergic neurons are needed.

## Data Availability Statement

The original contributions presented in the study are included in the article/Supplementary Material, further inquiries can be directed to the corresponding author.

## Ethics Statement

The animal study was reviewed and approved by Florida State University Animal Care and Use Committee.

## Author Contributions

GS, DG, DM, PB, and GL designed the research. GL, DG, BN, LA, and TT performed the research. GL, DG, GS, DM, BN, TT, and LA analyzed data. GL, DG, and GS wrote the manuscript. DM, PB, BN, LA, and MR provided edits to the manuscript. All authors contributed to the article and approved the submitted version.

## Conflict of Interest

The authors declare that the research was conducted in the absence of any commercial or financial relationships that could be construed as a potential conflict of interest.

## Publisher’s Note

All claims expressed in this article are solely those of the authors and do not necessarily represent those of their affiliated organizations, or those of the publisher, the editors and the reviewers. Any product that may be evaluated in this article, or claim that may be made by its manufacturer, is not guaranteed or endorsed by the publisher.

## Supplementary Materials

Supplementary Figure 1 Regional description of recombination patterns. *Emx1*-Cre and *Nkx2.1*-Cre mice were bred to the B6.Cg-Gt(ROSA)26Sortm14(CAG-tdTomato)Hze line. Offspring express bright tdTomato fluorescence throughout each cell where Cre-mediated recombination has occurred (red). Images were collected with a 2X objective and scale bar = 300 μm. The result of *Emx1* induced recombination is shown at the levels of the rostral striatum and nucleus accumbens **(A)**, mid-striatum **(B)**, and hippocampus **(C)**. Note that recombination is limited to the telencephalon. Nkx2.1-mediated recombination is also shown at the levels of the rostral striatum and nucleus accumbens **(D)**, mid-striatum **(E)**, and hippocampus **(F)**. Note the scattered cells throughout the cerebral cortex, but also positive cells within the striatum, basal forebrain, hypothalamus, and hippocampus, consistent with known expression patterns of Nkx2.1.

Supplementary Figure 2 Additional Phenotyping of Nkx2.1-derived neuronal subpopulations. Offspring from *Nkx2.1*-Cre and B6.Cg-Gt(ROSA)26Sortm14(CAG-tdTomato)Hze intercrosses were examined for additional markers. Images were collected with 20X objective and scale bar = 50 μm. Sections through the body of the striatum were immunostained with antibodies against DARPP-32 **(D)** or CHAT **(E)**. Note that Nkx2.1-recombined cells are DARPP32 negative, and some (but not all) are CHAT positive. Hippocampal recombined cells are present across multiple layers and subregions and are also DARPP-32 negative.
