# Supplementary figures and images for: Behavioral and Neuroanatomical Consequences of Cell-Type Specific Loss of Dopamine D2 Receptors in the Mouse Cerebral Cortex
Source: Front Behav Neurosci. 2022 Jan 13;15:815713. doi: 10.3389/fnbeh.2021.815713 (PMC8793809; doi:10.3389/fnbeh.2021.815713)

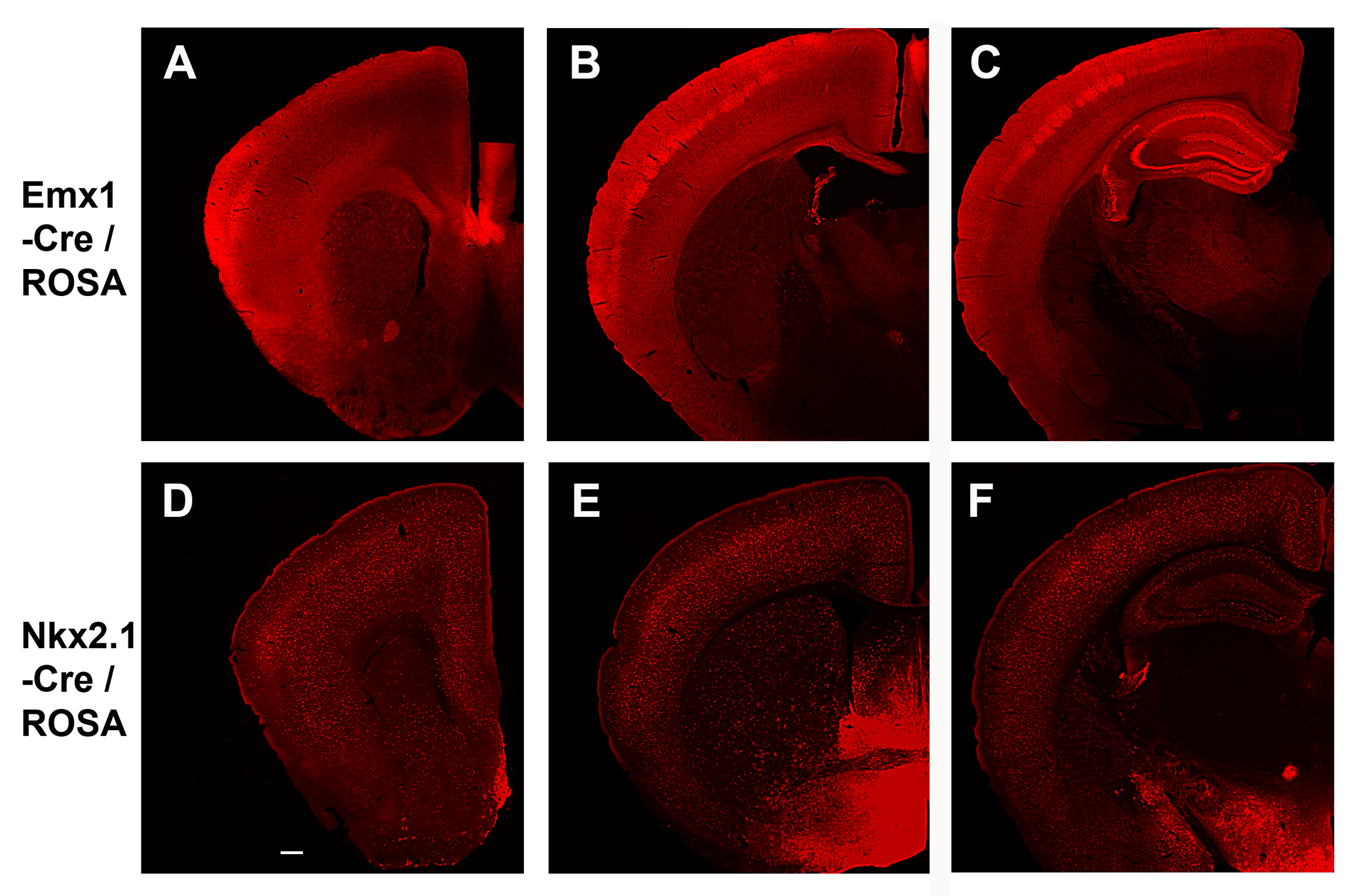

Supplement: Supplementary Figure 1 — Regional description of recombination patterns. Emx1-Cre and Nkx2.1-Cre mice were bred to the B6.Cg-Gt(ROSA)26Sortm14(CAG-tdTomato)Hze line. Offspring express bright tdTomato fluorescence throughout each cell where Cre-mediated recombination has occurred (red). Images were collected with a 2X objective and scale bar = 300 μm. The result of Emx1 induced recombination is shown at the levels of the rostral striatum and nucleus accumbens (A), mid-striatum (B), and hippocampus (C). Note that recombination is limited to the telencephalon. Nkx2.1-mediated recombination is also shown at the levels of the rostral striatum and nucleus accumbens (D), mid-striatum (E), and hippocampus (F). Note the scattered cells throughout the cerebral cortex, but also positive cells within the striatum, basal forebrain, hypothalamus, and hippocampus, consistent with known expression patterns of Nkx2.1. [file Image_1.TIF]

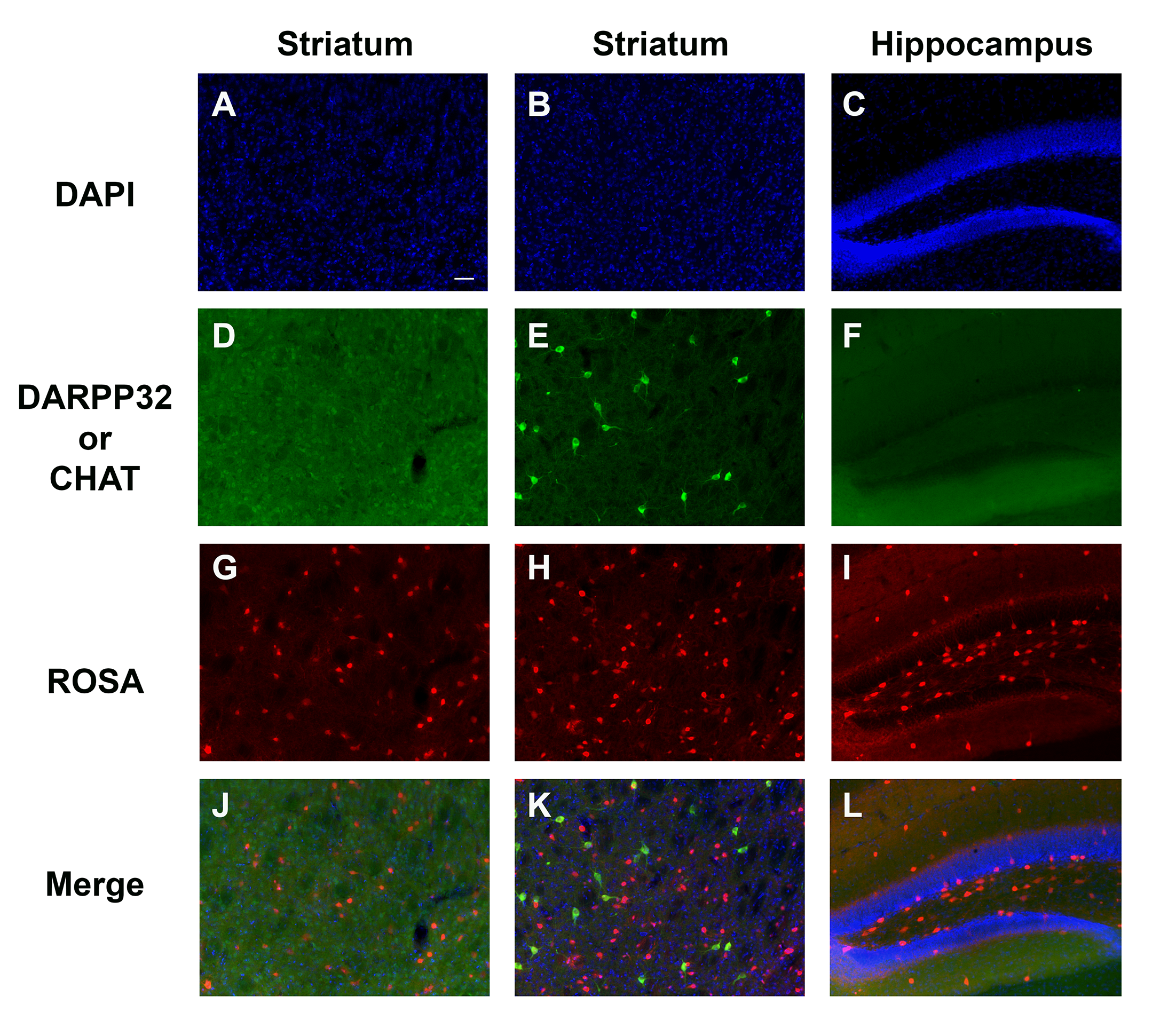

Supplement: Supplementary Figure 2 — Additional Phenotyping of Nkx2.1-derived neuronal subpopulations. Offspring from Nkx2.1-Cre and B6.Cg-Gt(ROSA)26Sortm14(CAG-tdTomato)Hze intercrosses were examined for additional markers. Images were collected with 20X objective and scale bar = 50 μm. Sections through the body of the striatum were immunostained with antibodies against DARPP-32 (D) or CHAT (E). Note that Nkx2.1-recombined cells are DARPP32 negative, and some (but not all) are CHAT positive. Hippocampal recombined cells are present across multiple layers and subregions and are also DARPP-32 negative. [file Image_2.TIF]
